# Autoimmune Neuromuscular Disorders at a Molecular Crossroad: Linking Pathogenesis to Targeted Immunotherapy

**DOI:** 10.3390/ijms262311736

**Published:** 2025-12-04

**Authors:** Anca-Maria Florea, Dimela-Gabriela Luca, Eugenia Irene Davidescu, Bogdan-Ovidiu Popescu

**Affiliations:** 1 Department of Clinical Neurosciences, “Carol Davila” University of Medicine and Pharmacy, 050474 Bucharest, Romania; anca-maria.florea@rez.umfcd.ro (A.-M.F.); bogdan.popescu@umfcd.ro (B.-O.P.); 2 Department of Neurology, Colentina Clinical Hospital, 020125 Bucharest, Romania; dimela_luca@yahoo.com; 3 Department of Cell Biology, Neurosciences and Experimental Myology, “Victor Babes” National Institute of Pathology, 050096 Bucharest, Romania

**Keywords:** myasthenia gravis, chronic inflammatory demyelinating polyneuropathy, idiopathic inflammatory myopathies, autoantibodies, targeted immunotherapy, complement inhibition, FcRn antagonists, B-cell depletion therapy

## Abstract

Myasthenia gravis, chronic inflammatory demyelinating polyneuropathy, and idiopathic inflammatory myopathies are among the most widely recognized autoimmune neuromuscular disorders. Although they differ in clinical presentation, shared immunopathogenic mechanisms place them at a molecular crossroads. Evidence of overlapping pathways has led to the development of targeted strategies including complement inhibition, FcRn antagonism, B-cell depletion, and the CAR-T cell approach. In this review, we analyze current knowledge regarding pathogenic mechanisms and their link to immunotherapy, extensively outlining both similarities and distinctions. We further discuss existing challenges, including diagnostic limitations and refractory disease variants, how technological advances have already addressed some of these issues, and where further progress is still needed.

## 1. Introduction

More than 600 distinct neuromuscular disorders have been identified and are typically classified by anatomical site of involvement, such as motor neurons, peripheral nerves, neuromuscular junctions, or muscle fibers, or by their underlying pathophysiology [1]. Autoimmune-mediated conditions constitute a major subgroup and are the primary focus of this review. The incidence and prevalence have increased over the past decade, a trend attributed to improved diagnostic methods, advances in therapy, heightened clinical awareness, and demographic changes associated with an aging population [2,3,4,5].

Despite clinical heterogeneity, autoimmune neuromuscular disorders share common immunological mechanisms. These convergent pathways establish a molecular framework for understanding disorders such as myasthenia gravis, chronic inflammatory demyelinating polyneuropathy, and idiopathic inflammatory myopathies, which are the primary focus of this review.

Therapeutic advances have paralleled mechanistic insights in this field. Molecularly targeted therapies, including complement inhibitors, neonatal Fc receptor (FcRn) antagonists, and B-cell-directed agents, are reshaping management strategies. Concurrently, technological innovations such as machine learning and advanced imaging are enhancing diagnostic precision and patient stratification.

This review aims not only to summarize disease-specific mechanisms but also to highlight the intersection between pathogenesis and targeted immunotherapy. By examining overlapping immune pathways and their therapeutic implications, the review clarifies how mechanistic insights have informed treatment advances and where further progress is needed.

A comprehensive literature search was performed using Google Scholar, PubMed, and Scopus. The search was limited to English-language publications, primarily from 2018 to 2025. Both studies addressing these disorders collectively and those examining each entity in detail were included. Additionally, the reference lists of selected articles were reviewed to identify further relevant sources.

## 2. Disease Overviews and Integrated Immunopathogenesis

**Figure 1:**
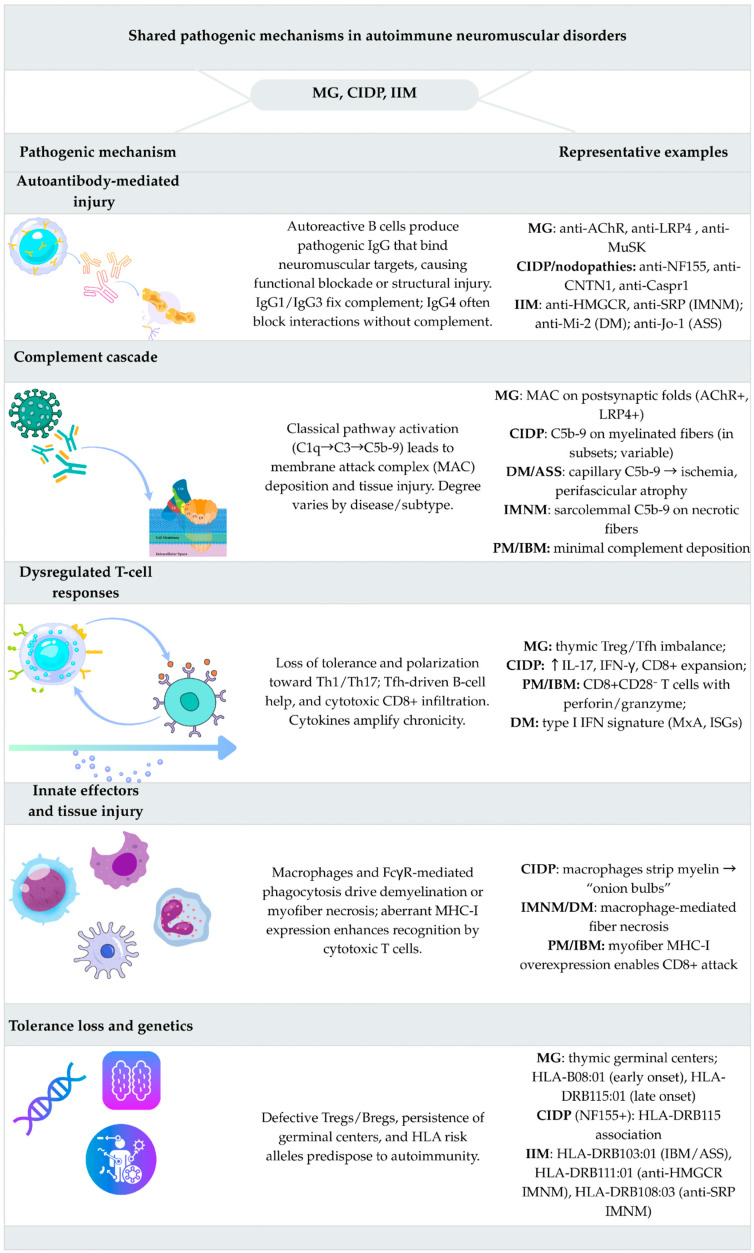
*Shared pathogenic mechanisms in autoimmune neuromuscular disorders.* The schematic highlights five convergent immunopathogenic pathways: autoantibody-mediated injury, complement activation, dysregulated T-cell responses, innate effector-driven tissue injury, and tolerance loss with genetic susceptibility- with representative examples from myasthenia gravis (MG), chronic inflammatory demyelinating polyneuropathy (CIDP), and idiopathic immune myopathies (IIM).

Myasthenia gravis (MG), chronic inflammatory demyelinating polyneuropathy (CIDP), and idiopathic inflammatory myopathies (IIM) exhibit overlapping effector mechanisms involving both adaptive and innate immune responses. However, each disorder possesses distinct molecular characteristics that define its clinical phenotype. Figure 1 provides a schematic overview of these shared immunopathogenic processes and highlights five principal mechanisms, with representative examples from each disease.

### 2.1. Myasthenia Gravis

#### 2.1.1. Clinical Features and Subtypes

Myasthenia gravis (MG) is a well-characterized autoimmune disorder that impairs neuromuscular transmission. It is clinically defined by fluctuating muscle weakness that worsens with sustained or repetitive activity and improves with rest [6,7]. Diagnosis is based on serological, electrophysiological, imaging, and functional assessments. Pathogenic autoantibodies most commonly target the acetylcholine receptor (AChR), muscle-specific kinase (MuSK), or low-density lipoprotein receptor-related protein 4 (LRP4). Agrin is less frequently implicated. Approximately 75–80% of patients have detectable AChR antibodies, 5–10% have MuSK antibodies, and LRP4 antibodies occur in a subset of previously labeled seronegative cases [8].

Anti-AChR-positive MG is classified as early-onset and late-onset forms, corresponding to onset in the third and sixth decades of life, respectively. Early-onset MG is associated with thymic hyperplasia containing germinal centers and increased expression of CXCL13, CCL21, and B-cell-activating factor (BAFF). In contrast, late-onset MG is linked to thymic atrophy. Anti-titin and anti-ryanodine receptor antibodies are present in late-onset MG [7]. Germinal center counts do not significantly differ between early- and late-onset patients. Treatment response does not correlate with thymic hyperplasia [7]. Both epigenetic and genetic factors contribute to these phenotypes and both subtypes share certain molecular features, including elevated serum levels of miRNA-150-5p and miRNA-21-5p. However, important distinctions remain. Next-generation sequencing has identified HLA-B*08:01 as the primary genetic risk factor for early-onset MG. HLA-DRB1*15:01 is most strongly associated with late-onset MG. Additionally, non-HLA loci differ. PTPN22 and TNFAIP3 are associated with early-onset MG and ZFAT variants are linked to late-onset disease [9].

There is a bidirectional association between thymoma and MG. Approximately 10 to 20 percent of MG patients have concomitant thymoma and roughly twice as many thymoma patients develop MG symptoms [9]. Loss of the AutoImmune Regulator (AIRE) protein is a proposed mechanism; AIRE is essential for the negative selection of autoreactive T cells. The autoantibody response often shifts from solely targeting AChR to also including titin, ryanodine receptor, and neurofilament [7].

Up to 85 percent of myasthenia gravis cases initially present with ocular symptoms. Ocular myasthenia gravis is defined by ptosis or diplopia due to weakness of the orbicularis oculi or extraocular muscles [10]. While ocular symptoms may remain isolated, many cases of ocular myasthenia gravis transition to a generalized disease. Both thymic hyperplasia and atrophy have been observed in these patients. HLA-DQ and PTPN22 variants influence disease risk, and miRNA 30-e-5P has emerged as a potential biomarker for predicting disease generalization [7,9].

Another important subtype is seen in immune checkpoint inhibitor-associated MG, which emerges in patients receiving specific cancer therapies. This subtype is defined by rapidly progressive, severe, and generalized muscle weakness. The severity often exceeds that observed in classical MG among patients receiving anti-cytotoxic T-lymphocyte-associated protein 4 (CTLA-4) and anti-programmed cell death protein 1 (PD-1) therapies [7].

#### 2.1.2. Immunopathogenesis

Antibody subclasses and effector pathways. Autoantibodies impair neuromuscular transmission through several mechanisms. These include complement-mediated postsynaptic injury, accelerated acetylcholine receptor (AChR) internalization, or direct disruption of receptor clustering or signaling [8]. In AChR-positive MG, immunoglobulin G1 (IgG1) and IgG3 antibodies activate the classical complement pathway. This results in membrane attack complex (MAC) deposition, loss of postsynaptic folds, and reduced functional AChRs. Clinically, this manifests as fatigable weakness [8,11]. In contrast, muscle-specific kinase (MuSK)-MG is primarily mediated by IgG4 antibodies. These become functionally monovalent by Fab-arm exchange. They inhibit low-density lipoprotein receptor-related protein 4 (LRP4)-MuSK-agrin signaling in a complement-independent manner, thus disrupting AChR clustering [8,12]. Anti-LRP4 autoantibodies, mainly IgG1 or IgG2, can activate complement and block agrin-LRP4-MuSK signaling. Passive-transfer models support their pathogenicity [11]. Notably, AChR- and LRP4-specific antibodies differ in their ability to activate the complement pathway. An overview of these autoantibodies and their involvement in myasthenia gravis pathogenesis is presented in Table 1.

Complement pathway. IgG1 and IgG3 antibodies bound at the neuromuscular junction recruit C1q, initiating the classical pathway activation. This process leads to C3b and C4b opsonization, release of anaphylatoxin C3a and C5a, and assembly of the MAC, which damages postsynaptic folds [13,14]. Anti-LRP4 antibodies are less effective at activating complement. Consistent with this, patients with anti-LRP4 MG exhibit lower circulating complement fragments. They often present with a milder phenotype compared to complement-driven anti-AChR MG [7,15]. The neonatal Fc receptor (FcRn) extends the half-life of IgG, maintaining the pool of pathogenic antibodies [7].

Immunoregulatory networks. Multiple regulatory cell populations contribute to the loss of immune tolerance in MG. In AChR-MG, impaired regulatory T cells (Tregs and T follicular regulatory cells, Tfr), reduced regulatory B cells (Bregs), and diminished myeloid-derived suppressor cell (MDSC) function promote T follicular helper (Tfh) cell-driven germinal center activation and production of IgG1 and IgG3 antibodies [16]. In MuSK-MG, defects in the tolerance checkpoint of naïve B cells and an altered memory B-cell repertoire predominate. There is also an expansion of short-lived plasmablasts producing pathogenic IgG4. An imbalance between Tfh and Tfr cells further amplifies autoreactivity [16,17]. Similar regulatory deficits are presumed in LRP4-MG [18].

#### 2.1.3. Crossroads Within MG

In summary, the pathogenesis of MG involves several immunologic targets. Autoantibody-mediated complement injury occurs at the neuromuscular junction. Pathogenic IgG is maintained by the neonatal Fc receptor (FcRn), and dysregulated B- and T-cell responses, often with thymic abnormalities, contribute to muscle weakness. These mechanistic insights inform therapeutic strategies. They include complement inhibitor, accelerated IgG clearance through FcRn blockade, depletion of autoreactive B cells, and rebalancing of T-cell responses. Thymectomy is effective in selected cases.

### 2.2. Chronic Inflammatory Demyelinating Polyneuropathy (CIDP)

#### 2.2.1. Clinical Features and Subtypes

Chronic Inflammatory Demyelinating Polyneuropathy (CIDP) is an immune-mediated neuropathy characterized by electrophysiologic or pathologic evidence of demyelination. The typical form of CIDP follows a chronic course with at least two months that may be progressive, stepwise, or relapsing. Key clinical features are symmetric proximal and distal weakness, sensory involvement in at least two limbs, and reduced or absent tendon reflexes [19,20]. Diagnosis relies on clinical assessment, electrodiagnostic findings and supporting evidence.

The updated EAN/PNS criteria have replaced the term “atypical CIDP” with CIDP variants, encompassing five clinical forms, which include pure sensory CIDP, pure motor CIDP, focal CIDP, chronic immune sensory polyradiculopathy (CISP), distal acquired demyelinating symmetric neuropathy (DADS), and multifocal acquired demyelinating sensory and motor neuropathy (MADSAM/Lewis-Sumner syndrome) [21]. Additionally, the guidelines reclassify patients with antibodies against nodal or paranodal cell-adhesion molecules, such as neurofascin isoforms (NF155, NF140, NF186, and pan-neurofascin), contactin-1 (CNTN1), and contactin-associated protein 1 (Caspr1) as having autoimmune nodopathies [22]. A short depiction of these is presented in the table below—Table 2.

#### 2.2.2. Immunopathogenesis

Autoantibody subclasses and effector pathways. The role of B-cells and humoral immunity in CIDP remains incompletely defined [25,26]. Alterations in B-cell phenotypes have been observed. Autoantibodies targeting peripheral nerve proteins, including myelin protein zero (P0), have been identified by Western blot analysis and in animal models. Experiments using B-cell-deficient mice further support a pathogenic role for these antibodies in neuropathy [27]. Additional evidence includes the deposition of IgG and IgM on Schwann cell surfaces and compact myelin in peripheral nerves [26]. In typical CIDP, nodal and paranodal regions are usually spared, and it remains unresolved whether macrophage-mediated injury originates at the node or internode [25]. Several candidate antigens have been investigated: peripheral myelin protein 22 (PMP22), myelin-associated glycoprotein (MAG), E-cadherin, and myelin protein zero (MPZ), but none have been conclusively validated. In contrast, antibodies against NF155, NF140/NF186, pan-neurofascin, CNTN1, and Caspr1 characteristic of autoimmune nodopathies are typically of the IgG4 isotype [25,26]. These antibodies do not induce classical demyelination, but instead disrupt axoglial junctions at the node of Ranvier, resulting in conduction slowing that can progress to conduction block, and, ultimately, secondary axonal transection [28].

Complement pathway and blood-nerve-barrier. Histopathology reveals complement deposition on both demyelinated and non-demyelinated nerve fibers, a finding that supports the hypothesis that complement activation disrupts the blood-nerve barrier, thereby facilitating adaptive immune responses. In treatment-naïve CIDP patients, elevated levels of C5a and the terminal complement complex C5b-9 (TCC) have been detected, with concentrations correlating with disease severity [29].

Immunoregulatory networks. T-cell activation and the release of pro-inflammatory cytokines represent the best-documented aspects of the cellular immune response. Increased Th17 (IL-17) and Th1 (IFN-γ) are detected in serum and CSF. Although the precise contribution of IL-17 remains unclear, animal models support its role in disease pathogenesis [27]. CD8+ T cells are also implicated, often more abundant than CD4+ cells, and display oligoclonal expansion consistent with antigen-driven, MHC class I-restricted attacks on peripheral nerve components [27,30].

Macrophage-mediated demyelination is recognized as a hallmark mechanism in CIDP and has been observed in 23–40% of analyzed nerve samples [29]. In these cases, macrophages containing myelin debris are found within the Schwann cell basal lamina, where they actively invade and separate the myelin sheath lamellae [25]. This mechanism is further supported by in vivo rat models of experimental autoimmune neuritis (EAN), which demonstrates macrophage-driven focal demyelination of nerve fibers [29]. Chronic, recurrent demyelination followed by remyelination leads to the formation of characteristic “onion bulb” formations [25].

#### 2.2.3. Crossroads Within CIDP

Current evidence indicates that the pathogenesis of “classical” CIDP is primarily mediated by cellular immune mechanisms, with macrophage-mediated segmental demyelination serving as the pathological hallmark. This mechanism underlies the generally favorable response to broad immunomodulatory therapies, including intravenous immunoglobulin (IVIG), corticosteroids, and plasma exchange. In contrast, patients with IgG4 paranodal autoantibodies (e.g., CNTN1, Caspr1, NF155) are now classified as having autoimmune nodopathies. In these cases, antibodies disrupt nodal and paranodal adhesion rather than inducing typical demyelination, which is frequently associated with poor response to IVIG but improved outcomes with B-cell-depleting therapies. Although complement deposition and humoral factors are inconsistently observed in classical CIDP, the clinical efficacy of therapies that reduce IgG levels or inhibit complement in selected patients suggests that circulating immune factors contribute to pathogenesis in at least a subset of cases. This continuum, ranging from predominantly cellular demyelinating disease to antibody-mediated nodal pathology, supports therapeutic strategies that span broad immunosuppression to more targeted interventions, including FcRn blockade, complement inhibition, and B-cell modulation, as discussed in subsequent sections.

### 2.3. Idiopathic Inflammatory Myopathies (IIM)

**Figure 2:**
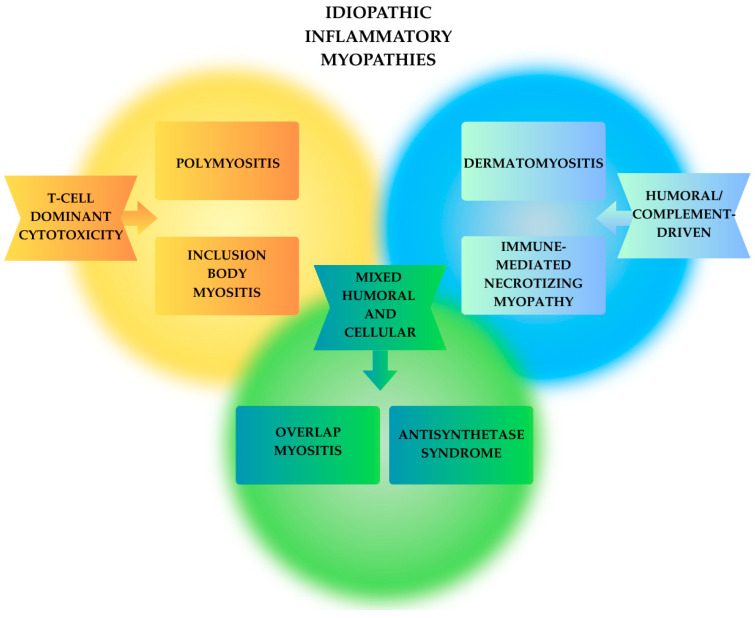
*Main immunopathogenic drivers of idiopathic inflammatory myopathies*. Subtypes are grouped by their predominant immune drivers: T-cell dominant cytotoxicity (polymyositis, inclusion body myositis), humoral/complement-driven injury (dermatomyositis, immune-mediated necrotizing myopathy), mixed humoral and cellular mechanisms (overlap myositis, antisynthetase syndrome).

Idiopathic inflammatory myopathies comprise a group of autoimmune muscle disorders defined by chronic muscle inflammation, weakness, and frequent multisystem involvement. The original Bohan and Peter (1975) [31] categories: polymyositis (PM), dermatomyositis (DM), and inclusion body myositis (IBM), have been refined through the identification of myositis-specific autoantibodies (MSAs), advances in histopathology, and outcomes research. The 2017 EULAR/ACR criteria enhanced classification but did not fully distinguish emerging subsets, necessitating ongoing refinements [31,32]. Idiopathic inflammatory myopathies (IIM) can be classified according to key pathogenic mechanisms: humoral/complement-dominant entities (such as dermatomyositis and immune-mediated necrotizing myopathy), prototypical T-cell-dominant forms (polymyositis), and inclusion body myositis, which combines chronic CD8+ cytotoxicity with degenerative protein-aggregation pathology. This conceptual heterogeneity, driven by the dominant immunopathogenic drivers, is summarized in Figure 2 and specifically described in the following sections.

#### 2.3.1. Dermatomyositis (DM)

##### Clinical Features and Subtypes

Dermatomyositis (DM) is characterized by distinctive cutaneous lesions such as heliotrope rash, Gottron papules, scaly lesions over bony prominences, the shawl sign, and the V-sign, along with symmetric proximal muscle weakness. Extramuscular manifestations include interstitial lung disease, dysphagia, and arthritis or Raynaud’s phenomenon [33].

A 2019 consensus identified six MSA-defined subtypes. These are anti-NXP2, anti-Mi2, anti-MDA5, anti-SAE, and anti-TIF1-γ. Each subtype has distinct diagnostic and prognostic implications. The DM spectrum also includes amyopathic and hypomyopathic forms, which are frequently associated with specific antibodies [34].

##### Immunopathogenesis

Autoantibody subclasses and effector pathways. Myositis-specific autoantibodies (MSAs) are present in approximately 80–90% of patients. They correspond to specific clinical and pathological features. Antibodies against Mi-2 are associated with classic cutaneous and muscular findings. Anti-TIF1-γ (also known as anti-p155/140) is frequently linked to malignancy, anti-MDA5 is strongly associated with clinically amyopathic DM and interstitial lung disease (ILD), anti-NXP2 and anti-SAE1/2 with specific histologic patterns [35]. Histopathological findings reflect these subtypes: anti-TIF1-γ, anti-NXP2, and anti-SAE antibodies are associated with perifascicular atrophy (PFA), anti-Mi-2 with perifascicular necrosis (PFN) and inflammation, and anti-MDA5 with milder, nonspecific myopathic changes [36].

Complement pathway. Complement activation leads to the early deposition of membrane attack complex (MAC, C5b-9) on endothelial cells. This results in capillary injury within the endomysium with subsequent ischemia and perifascicular microinfarcts. These events underlie the development of perifascicular atrophy (PFA). Such observations have been incorporated into diagnostic histopathological criteria, alongside MxA expression [36,37].

Immunoregulatory networks. T-cells also contribute to tissue injury. CD4+ and CD8+ T-cell populations are present within affected tissues, with a predominance of CD4+ T cells in muscle biopsies. Clonally expanded cytotoxic T cells persist in muscle, even in patients treated with immunosuppression, suggesting a role in disease chronicity [38].

Type-I interferon signaling. Overexpression of interferon-stimulated genes (ISGs) is consistently observed in muscle and skin, while serum IFN-α and IFN-β levels correlate with disease activity. Myxovirus resistance protein A (MxA), an IFN-I-induced marker, localizes to myofibers and small vessels within perifascicular regions [36]. Monoclonal antibodies derived from B cells can also enhance IFN-γ release, supporting their direct pathogenic role [35].

Genetic predisposition modulates disease risk in DM. Specific HLA alleles influence antigen presentation and shape immune responses, contributing to clinical heterogeneity [35].

##### Crossroads Within DM

Dermatomyositis represents a humoral-dominant idiopathic inflammatory myopathy, defined by autoantibody stratification and complement-dependent vascular injury.

#### 2.3.2. Polymyositis (PM)

##### Clinical Features and Subtypes

Polymyositis (PM) is now recognized as a rare phenotype. It is primarily a diagnosis of exclusion after other idiopathic inflammatory myopathies are considered [39]. Clinically, PM presents with subacute, symmetric proximal or truncal weakness and elevated muscle enzymes. Myopathic electromyography findings and supportive histology are also present, but the characteristic dermatomyositis rash is absent [40]. Definitive diagnosis requires muscle biopsy evidence of endomysial inflammation surrounding or invading non-necrotic fibers [41].

##### Immunopathogenesis

Autoantibody subclasses and effector pathways. In contrast to other idiopathic inflammatory myopathies, PM lacks a defining myositis-specific autoantibody profile. Humoral involvement is minimal; plasma cells and clonally expanded B cells are occasionally detected in endomysial infiltrates, but their contribution is secondary to T-cell-mediated cytotoxicity [42].

Complement pathway. Complement deposition is not a prominent feature of PM. Unlike other myopathies, muscle fiber necrosis in PM is not primarily mediated by antibody or complement, reinforcing PM as a T-cell-driven disease [42].

Immunoregulatory networks. PM is primarily driven by antigen-specific CD8+ T cells that target myofibers aberrantly overexpressing MHC-I [42]. Immunoelectron microscopy reveals CD8+ T cells and macrophages extending spike-like processes through the basal lamina into intact fibers, with perforin- and granzyme-rich granules directed toward the sarcolemma, including areas of necrosis. Elevated IFN-γ and TNF-α promote aberrant MHC-I upregulation, often preceding visible inflammation [42].

Antigen specificity. Clonal T-cell receptor rearrangements in endomysial CD8+ T cells, along with local chemokine, cytokine receptor and matrix metalloproteinases expression, support the presence of an antigen-driven immune synapse. Adhesion and extracellular matrix molecules (thrombospondins, ICAM, VCAM) enhance leukocyte recruitment and retention. Myeloid dendritic cells, potent antigen-presenting cells, are abundant in infiltrates, while plasma cells and clonally expanded B cells further implicate the adaptive immune system in the inflammatory network [42].

Genetic and transcriptional profiles indicate a predominance of CD8+ T cells in PM, in contrast to the relative abundance of CD4+ T cells, plasmacytoid dendritic cells, and B cells in DM. RNA sequencing of peripheral T cells reveals broadly similar profiles across idiopathic inflammatory myopathies, but differential analysis identifies 176 CD8+ T cell genes that distinguish PM, many of which are linked to lymphocyte migration and T-cell differentiation [43].

##### Crossroads Within PM

Polymyositis is characterized by T-cell dominance, specifically CD8+ cytotoxic T-cell activity against MHC-I overexpressing muscle fibers.

#### 2.3.3. Immune-Mediated Necrotizing Myopathy (IMNM)

##### Clinical Features and Subtypes

Immune-mediated necrotizing myopathy is characterized by myofiber necrosis with minimal lymphocytic infiltrates, markedly elevated serum creatine kinase, and rapidly progressive, predominantly proximal muscle weakness [44].

Recognized as a distinct entity by the ENMC in 2004 [45], IMNM includes two principal subtypes: anti-SRP, which targets the signal recognition particle, and anti-HMGCR, which targets 3-hydroxy-3-methylglutaryl-CoA reductase [44]. Anti-HMGCR-positive IMNM frequently follows statin exposure but can also occur in statin-naive patients, including children [46]. Clinically, anti-SRP IMNM is associated with an increased risk of myocarditis, while seronegative IMNM has a higher association with malignancy [44]. Muscle biopsy typically reveals necrosis and regeneration disproportionate to inflammation, variable MHC-I upregulation, patchy sarcolemma C5b-9 deposition, and frequent p62 positivity [47].

##### Immunopathogenesis

Autoantibody subclasses and effector pathways. The signal recognition particle (SRP) is an intracellular ribonucleoprotein complex. Approximately 80% of anti-SRP patients have antibodies against SRP54, especially its N-terminal or G-central region, with minimal reactivity to the C-terminal region [48,49]. 3-hydroxy-3-methylglutaryl-coenzyme A Reductase (HMGCR) is an endoplasmic reticulum resident enzyme. Mapping studies identify the C-terminal portion as the principal epitope [49,50]. Both SRP and HMGCR can be detected on the sarcolemma of injured or regenerating fibers, providing accessible targets for circulating antibodies [51]. Autoantibody binding can impair myoblast differentiation and fusion [44], associated with reduced levels of the key cytokines for myoblast function, IL-4 and IL-13 [44,52,53]. Beyond immune injury, antibody exposure also triggers non-immune catabolic pathways, upregulating the E3 ubiquitin-protein ligase TRIM63 (MuRF1) and muscle atrophy F-box protein (MAFbx/atrogin 1) and thereby accelerating muscle loss [44,54].

Complement pathway. Antibody binding to these ectopically displayed antigens can be followed by classical complement activation, from C1q engagement to C5b-9 (MAC) assembly, culminating in myofiber necrosis. Even with minimal complement activity, antibody ligation induces oxidative and cellular stress [47,51].

Immunoregulatory networks. Despite paucicellular histology, recent biopsy studies have delineated a T-cell component. HMGCR-specific CD4+ T cells have been detected in both blood and affected muscle, supporting an active pathogenic role. The response is Th1/Th17-polarized, and its magnitude correlates positively with anti-HMGCR IgG levels [55]. Direct cytotoxic infiltration of non-necrotic fibers is uncommon; instead, inhibitory-receptor signaling (PD-1, PD-L1, PD-L2, TIM-3) suggests functional exhaustion, though the therapeutic relevance of PD-1 modulation remains uncertain [56].

Macrophages. Biopsies reveal a macrophage-rich infiltrate, consistent with antibody- and complement-mediated injury and Fc receptor-dependent myophagocytosis. Macrophages contribute to opsonization and promote fiber necrosis [51]. Transcriptomic and immunohistochemical evidence indicates a Th1-driven pro-inflammatory profile in IMNM, characterized by STAT1+/CD68+ and CD68+/NOS2+ macrophages invading myofibers, supporting macrophage-mediated myophagocytosis as a key pathogenic mechanism [51].

Genetics. Distinct HLA associations have been identified across IMNM subtypes. In anti-HMGCR IMNM, HLA-DRB*07:01 is linked to pediatric cases and HLA-DRB1*11:01 to statin-naïve adults [57]. In anti-SRP IMNM, HLA- DRB1*08:03 is a major risk allele in Japanese patients; HLA-DRB1*14:03 has been reported in Koreans, while in African Americans DQA1*0102 and DRB1*08 are enriched among anti-SRP positive cases [57,58,59].

Myofiber-intrinsic stress pathways. In addition to inflammatory and humoral mechanisms, non-immune pathways, particularly those intrinsic to myofibers, are increasingly recognized in IMNM. Experimental overexpression of MHC class I on myofibers in mice induces endoplasmic reticulum (ER) stress with glucose-regulated protein 78 (GRP78/BiP) upregulation [60]. Autophagy-lysosome activation (LC3, LAMP2, acid phosphatase), mitophagy (in HMGCR-IMNM), and necroptotic signaling (in SRP-IMNM) further contribute to fiber death. Together, these non-immune stress pathways integrate with antibody/complement injury to perpetuate muscle damage and impair effective repair [60].

##### Crossroads Within IMNM

Immune-mediated necrotizing myopathy is an idiopathic inflammatory myopathy driven by antibody and complement activity, with additional amplification from macrophage-mediated necrosis and intrinsic myofiber stress pathways.

#### 2.3.4. Inclusion Body Myositis (IBM)

##### Clinical Features and Subtypes

Inclusion body myositis (IBM) most often manifests after the age of fifty with a gradual onset, predominant distal muscle weakness, and a slow progression. The current EULAR/ACR criteria highlight key features, including finger-flexor weakness and the presence of vacuolated muscle fibers [34,61].

##### Immunopathogenesis

Autoantibody subclasses and effector pathways. While IBM is primarily characterized by T-cell dominance, humoral immunity also plays a significant role in disease pathogenesis. CD20+ B cells are infrequent in muscle tissue, but clonal differentiation into CD19+ plasmablasts and CD138+ plasma cells have been documented. This process has facilitated the identification of anti-cN1A (anti-cytosolic 5′-nucleotidase 1A) autoantibodies, which are detected in a subset of patients but do not demonstrate complete sensitivity and specificity [61].

Complement pathway. Unlike in dermatomyositis, where complement activation is central to pathogenesis, complement deposition is not a characteristic feature of IBM. However, isolated deposits may occasionally be reported, particularly in cases involving overlap syndromes or diagnostic uncertainty [62].

Immunoregulatory networks. A defining feature of IBM is the infiltration of non-necrotic myofibers by cytotoxic CD8+ T cells, which differentiates it from polymyositis. Endomysial display a fivefold predominance of CD8+ over CD4+ T cells, with clonal expansion detected in both muscle and peripheral blood, indicating antigen-driven responses. These T cells possess a late effector phenotype, marked by the proliferation of effector memory T cell and terminally differentiated effector memory T cells. CD8+CD28-T cells produce high levels of IFN-γ and express high levels of granzymes and perforin, consistent with natural killer cell-like cytotoxicity. Additionally, myofiber injury may occur independently of classical TCR/CD3 recognition and CD28 co-stimulation, suggesting the involvement of innate-like cytotoxic mechanisms [61].

Genetics. Genetic susceptibility to IBM is linked to HLA-DRB1*03:01, HLA-B*08:01, and specific polymorphisms in CCR5 gene [61].

Degenerative and non-immune mechanisms. IBM is characterized by degenerative and immune-mediated injury. Affected muscle display rimmed vacuoles, cytoplasmic protein aggregates, and mitochondrial abnormalities, all indicative of impaired proteostasis. These aggregates, which include ubiquitin, tau, amyloid, and β-amyloid, are associated with endoplasmic reticulum (ER) stress and defective autophagy [61]. Such features, together with mitochondrial dysfunction and abnormal protein homeostasis, contribute to myofiber damage. Additional pathological markers include the accumulation of p62, TDP-43, and LC3, which further highlight ER stress, altered autophagy, and the mislocalisation of myonuclear heterogenous nuclear ribonucleoproteins [61].

##### Crossroads Within IBM

Inclusion body myositis is defined by persistent, antigen-driven CD8+ T-cell cytotoxicity and degenerative muscle pathology, including rimmed vacuoles, cytoplasmic protein aggregates such as p62, TDP-43, β-amyloid, and ubiquitin, as well as mitochondrial abnormalities and impaired autophagy. The simultaneous presence of immune and degenerative mechanisms likely accounts for the limited efficacy of conventional immunosuppressive treatments and therapies that address only individual degenerative pathways.

#### 2.3.5. Antisynthetase Syndrome (ASyS)

##### Clinical Features and Subtypes

Antisynthetase syndrome (ASyS), now widely recognized as a distinct idiopathic inflammatory myopathy, is characterized by the presence of anti-tRNA synthetase antibodies in conjunction with myositis and/or extramuscular manifestations, such as arthritis, “mechanic’s hands”, Raynaud’s phenomenon, fever, or interstitial lung disease [63]. The ENMC dermatomyositis workshop also recommends considering ASyS in patients who present with cutaneous manifestations [64].

##### Immunopathogenesis

Autoantibody subclasses and effector pathways. The most frequent antibody is anti-Jo-1, with the additional hallmark antibodies: anti-PL-7, anti-PL-12, anti-EJ, anti-OJ, anti-KS, anti-Zo, and anti-Ha. These antibodies, detected in approximately 20–30% of patients with idiopathic inflammatory myopathies, target core translational enzymes [36]. Anti-Jo-1 is common and overlaps with DM features, particularly antibody- and complement-mediated microangiopathy [36].

Complement pathway. The interaction of Jo-1 antibodies with capillary antigens triggers the deposition of complement, leading to endothelial injury, ischemia, and perifascicular necrosis (PFN). This pathological pattern is similar to that observed in anti-Mi-2 positive DM, but it does not occur in all DM subtypes [36].

Immunoregulatory networks. Muscle biopsy findings in ASyS typically demonstrate T-cell and macrophage infiltration, along with perifascicular atrophy or necrosis. Without serological testing, ASyS can be misclassified as polymyositis (PM), immune mediated necrotizing myopathy (IMNM), or dermatomyositis (DM) [36]. MHC-I upregulation is prominent, with variable MHC-II expression. Complement-mediated myofiber death is less pronounced than in DM, highlighting both shared and distinct pathogenic pathways [64].

##### Crossroads Within ASyS

ASS integrates humoral and cellular mechanisms, involving antibody- and complement-mediated vascular injury, as well as T-cell and macrophage infiltration.

#### 2.3.6. Overlap Myositis (OM)

##### Clinical Features and Subtypes

Overlap myositis (OM) is a type of inflammatory myopathy that occurs along with other connective tissue diseases, including systemic lupus erythematosus, Sjogren’s syndrome, systemic sclerosis, or rheumatoid arthritis [34]. While some experts group overlap myositis (OM) and antisynthetase syndrome (ASyS) together, others distinguish them based on clinical and serological differences [65].

##### Immunopathogenesis

Autoantibody subclasses and effector pathways. Common autoantibodies identified in OM are anti-PM (polymyositis)/Scl (scleroderma) and anti-U1 RNP (ribonucleoprotein). Anti-U1 RNP is particularly associated with pulmonary involvement [65].

Complement pathway. Histopathology analysis can demonstrate deposition of the membrane attack complex (MAC) on muscle fibers, although this finding is less consistent than in dermatomyositis. The role of complement is variable and not invariably pathogenic in OM [66].

Immunoregulatory networks. Muscle biopsies in OM often shows prominent lymphocytic infiltration, muscle fiber necrosis, and perivascular inflammation. Vasculitis is more common in OM than in other inflammatory myopathies. In chronic cases, endomysial fibrosis may appear. Immunohistochemical analysis usually reveals strong membranous and cytoplasmic HLA-ABC (MHC-I) expression. In contrast, HLA-DR expression is less often seen [66].

##### Crossroads Within OM

Overlap myositis exhibits a hybrid pattern that integrates connective tissue disease-associated autoantibodies with lymphocytic vasculitis and muscle inflammation. 

## 3. Molecular and Targeted Therapies

The convergence of therapeutic strategies across myasthenia gravis, chronic inflammatory demyelinating polyneuropathy, and idiopathic inflammatory myopathies directly impacts clinical decision-making, offering a well-structured framework for targeted treatment. Given the shared immunopathogenic mechanisms, numerous molecular targets and drug classes are utilized in multiple disorders. Positive outcomes in one context frequently prompt investigation in others. Table 3 categorizes both established and emerging therapies according to their principal immune targets, thereby illustrating the common therapeutic landscape and identifying future opportunities.

### 3.1. Myasthenia Gravis

**Figure 3:**
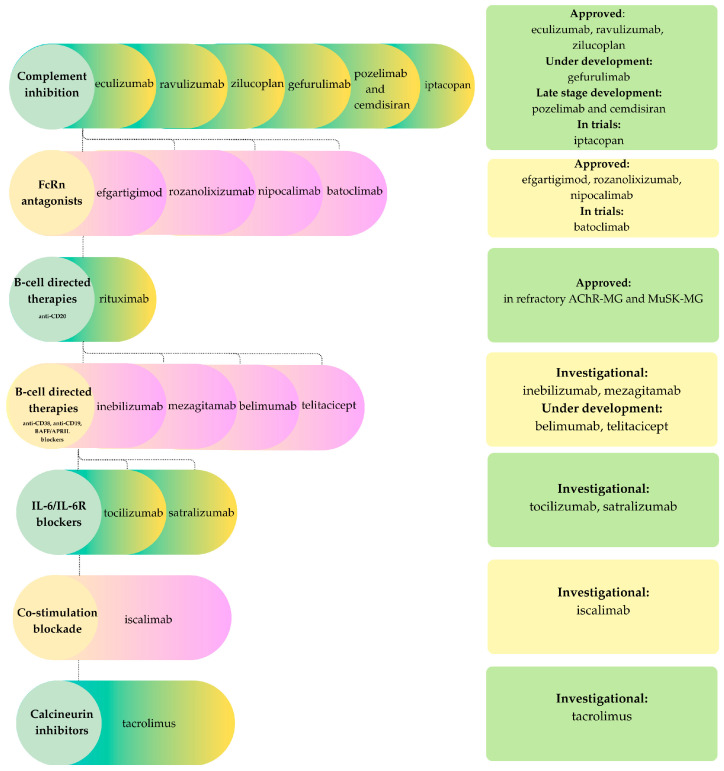
*Drug classes, corresponding representatives and approval status in myasthenia gravis.* Drug classes are presented with their corresponding representatives, while status boxes summarize which agents are approved for use in myasthenia gravis, which are still under development and which are investigational only; MG = myasthenia gravis, AChR = acetylcholine receptor, MuSK = muscle-specific tyrosine kinase, FcRn = neonatal Fc receptor, CD = cluster of differentiation, BAFF/APRIL = B-cell activating factor/A proliferation-inducing ligand, IL-6/IL-6R = interleukin-6, interleukin-6 receptor.

Therapeutic strategies for myasthenia gravis are most effectively conceptualized in relation to the disease’s dominant effector pathways. Complement inhibitors protect the neuromuscular junction from IgG1/IgG3-mediated complement injury and therefore demonstrate their greatest efficacy in AChR- and LRP4-positive MG, where classical pathway activation is central. In contrast, interventions that reduce circulating IgG, such as neonatal Fc receptor (FcRn) antagonists, or deplete B cells, target the autoantibody axis more broadly and can also benefit IgG4-mediated forms such as MuSK-positive MG. Broader immunosuppressive therapies directed at T and B cells modulate upstream autoreactive responses [64]. Figure 3 presents a schematic representation of the drug classes used in myasthenia gravis, with their corresponding representatives and approval status.

*1.*  *Complement inhibitors*
  As activation of the complement cascade is a key driver of pathology in AChR antibody-mediated MG and LRP4 MG, inhibiting the terminal pathway represents an evident therapeutic approach. Eculizumab, the first approved complement inhibitor (2017), remains a milestone therapy for refractory AChR-positive generalized MG. Ravulizumab, its long-acting successor, demonstrated compelling efficacy in the CHAMPION-MG Phase 3 trial and was FDA-approved in 2022. Zilucoplan, a subcutaneous macrocyclic peptide that blocks both C5 cleavage and C5b–C6 binding, showed rapid clinical benefit and gained approval in 2023 [64,67]. Additional candidates include gefurulimab, which inhibits hepatic complement synthesis, and the combination of pozelimab (anti-C5 antibody) with cemdisiran (a small interfering RNA that suppresses hepatic C5 production), currently in late-stage development [67]. Iptacopan, a factor B inhibitor of the alternative pathway, is also under evaluation, whereas vemircopan, a factor D inhibitor, was discontinued due to limited efficacy [67].
*2.*  *FcRn antagonists*
  The neonatal Fc receptor (FcRn) regulates immunoglobulin G (IgG) homeostasis. Inhibition of FcRn accelerates the degradation of IgG, thereby reducing both pathogenic and non-pathogenic antibodies. Efgartigimod, approved in 2021 following the Phase 3 ADAPT trial, was the first agent in this class to show marked IgG with significant clinical benefit in AChR-positive MG [67]. Rozanolixizumab received approval for both AChR-positive and MuSK-positive MG, demonstrating the broad applicability of FcRn blockade, which reduces IgG levels regardless of antigen specificity. This is particularly relevant in MuSK-MG, where IgG4 antibodies mediate disease independently of complement activation [67]. Nipocalomab, a next-generation aglycosylated monoclonal antibody, recently received FDA approval for generalized MG [68]. Batoclimab, a fully human IgG1 monoclonal antibody structurally modified to minimize cytotoxicity, is progressing through Phase 3 development. Across clinical studies, FcRn antagonists consistently achieve reductions exceeding 60% in total IgG within weeks, with corresponding clinical improvements in patients receiving immunotherapy [64]. Similar to complement inhibitors, these agents are expensive biologic therapies that necessitate repeated or prolonged administration to sustain IgG suppression and reduce both protective and pathogenic IgG. Clinical trials have reported infectious adverse events associated with their use. Consequently, these therapies are currently primarily reserved for patients exhibiting high disease activity or refractory myasthenia gravis [64,67,68].
*3.*  *B-cell and plasma-cell therapies*
  B cells and plasma cells contribute to the pathogenesis of myasthenia gravis by producing autoantibodies, making them important therapeutic targets. Rituximab, a chimeric mouse-human monoclonal antibody targeting the B cell surface antigen CD20, is utilized in refractory AChR-positive and MuSK-positive MG, with strong evidence supporting early intervention in MuSK-MG [64,67]. Additional B-cell-directed agents under active investigation include inebilizumab, a humanized anti-CD19 monoclonal antibody that broadly depletes CD19-expresing B cells, and mezagitamab, an anti-CD 38 monoclonal antibody targeting plasma cells, although efficacy has been modest. The B-cell activating factor (BAFF)/B-lymphocyte stimulator (BLyS) pathway is also a promising target. Belimumab, an anti-BAFF monoclonal antibody that inhibits B-cell proliferation and maturation, has demonstrated preliminary activity. Telitacicept, a fully human TACI-Fc fusion protein that neutralizes BAFF and BLyS, is in development for MG following its approval in systemic lupus erythematosus [67].
*4.*  *Other immunomodulators*
  Pro-inflammatory cytokines and T-B cell interactions contribute to the pathogenesis of myasthenia gravis; thus, interleukin-6 (IL-6) receptor represents a viable therapeutic strategy. Satralizumab, a humanized monoclonal antibody targeting the IL-6 receptor, is already approved for seropositive neuromyelitis optica spectrum disorder (NMOSD) and is under investigation for MG. Tocilizumab, another IL-6 receptor blocker, is in Phase 2 trials for generalized MG, with preliminary reports indicating positive effects in refractory cases [67,69]. T-cell directed strategies include iscalimab, a monoclonal antibody against CD40 that disrupts co-stimulation between T and B cells [67]. Tacrolimus, a calcineurin inhibitor, serves as a steroid-sparing agent and may further benefit patients by stabilizing acetylcholine receptor clustering at the neuromuscular junction [70,71].
*5.*  *Cell-based and novel therapies*
  In patients with myasthenia gravis who have persistent autoreactive B and T cells, cellular therapies such as chimeric antigen receptor T (CAR-T) cell therapies targeting B-cell markers or autologous hematopoietic stem cell transplantation are designed to restore immune tolerance. A completed Phase 2b trial of Descartes-08, an autologous RNA-based CAR-T therapy targeting B-cell maturation antigen (BCMA), is pending publication. Early data showed that repeated infusions without lymphodepleting chemotherapy were safe and well tolerated, with no dose-limiting toxicities, cytokine release syndrome, or neurotoxicity and only transient, mild adverse events [72]. Anti-CD19 CAR-T therapy, employing a DNA-based approach with lymphodepleting conditioning, has demonstrated promising outcomes in three reported refractory MG cases: one AChR-positive and two with concomitant Lambert-Eaton myasthenic syndrome (LEMS), achieving effective CD19-positive B-cell depletion and significant clinical improvement over two to six months of follow-up [73,74]. Together, these early data indicate that CAR-T-mediated depletion of pathogenic B cells and plasma cells can induce profound remission in highly refractory MG with an acceptable short-term safety profile in the reported series [72,73,74]. Another approach in development, MuSK-chimeric autoantibody receptor (CAAR-T) therapy, which uses chimeric autoantibody receptor T cells to selectively eliminate MuSK-specific B cells while sparing healthy B cells [67].
  Autologous hematopoietic stem cell transplantation (AHSCT) has been used in highly refractory MG, both AChR-positive or MuSK-positive MG, and even in seronegative patients. The majority of patients achieved complete remission or minimal manifestation status over a follow-up period of 1.5 to 10 years, often discontinuing all MG medications. A Phase 2 trial evaluating HSCT in MG is ongoing [65].
*6.*  *Surgical advances*
  Thymectomy removes the site of autoantibody initiation in AChR-MG and can induce long-term remission, making it a cornerstone of treatment. Clinical trials demonstrated its superiority over conventional immunosuppression alone, with sustained benefit at five years [66]. Minimally invasive and robotic techniques now provide comparable remission rates with reduced morbidity compared to open surgery [75].
*7.*  *Special considerations*
  Sex and age shape both the clinical presentation and prognosis of MG. Early-onset AChR-positive disease demonstrates a female predominance, while late-onset and thymoma-associated MG are more common in older men. Ocular presentations are particularly prevalent among elderly patients [64,76]. Currently, there is no targeted therapy specifically approved for ocular MG, and this subgroup is typically excluded from clinical trials of novel biologic agents. As a result, management primarily relies on symptomatic treatment and conventional immunotherapy, most often low-to-moderate dose corticosteroids with or without steroid-sparing agents. Observational studies indicate that early initiation of immunosuppression may reduce or delay secondary generalization in some cohorts; however, results are heterogenous and the quality of evidence remains limited. Consequently, this approach is based largely on expert consensus rather than high-level trial data [76]. Major randomized trials of complement inhibitors, FcRn antagonists and B-cell-directed agents in generalized myasthenia gravis have not identified significant sex-based differences in efficacy or safety. However, most studies lacked sufficient power for formal sex-stratified analyses.
  In seronegative MG, FcRn inhibitors may offer broad benefit regardless of antibody status, whereas complement inhibitors are unlikely to be helpful unless low-affinity AChR antibodies are present. Advanced assays can identify low-density AChR antibodies in some seronegative cases, who then respond similarly to seropositive patients. For truly seronegative disease, non-specific immunosuppression remains the mainstay, though biomarker-driven targeted therapy is a priority for future research [64,67,76].

### 3.2. CIDP

**Figure 4:**
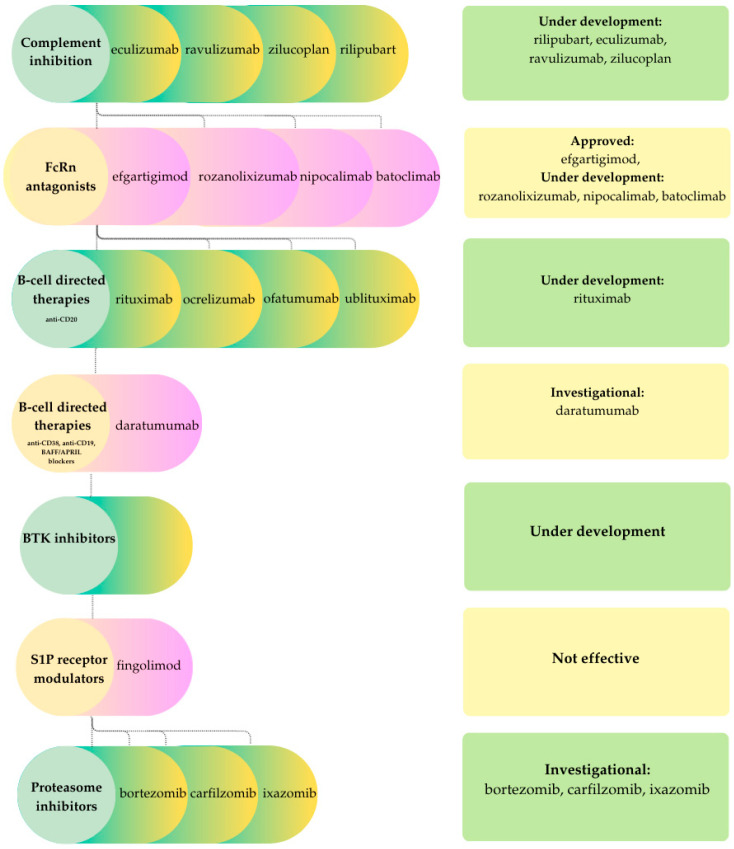
*Drug classes, corresponding representatives and approval status in CIDP.* Drug classes are presented with their corresponding representatives, while status boxes summarize which agents are approved for use in chronic inflammatory demyelinating polyneuropathy, which are still under development and which are investigational only; FcRn = neonatal Fc receptor, CD = cluster of differentiation, BAFF/APRIL = B-cell activating factor/A proliferation-inducing ligand, BTK = Bruton’s tyrosine kinase, S1P = sphingosine-1-phosphate.

Therapeutic strategies for chronic inflammatory demyelinating polyneuropathy (CIDP) must address a pathogenic spectrum that extends from predominantly cellular, macrophage-mediated demyelination in classical forms to antibody-driven disruption of the node/paranode in autoimmune nodopathies. Broad immunomodulatory therapies such as corticosteroids, intravenous or subcutaneous immunoglobulin, and plasma exchange remain first-line options and are often effective in typical CIDP. However, these treatments are associated with adverse effects, substantial costs, and only partial or inconsistent benefit in certain patients, particularly those with IgG4 paranodal antibodies [77,78]. These findings have led to a transition toward more mechanism-based interventions, including complement inhibition and FcRn blockade to target pathogenic IgG and its effector pathways, as well as B-cell-directed therapies for antibody-mediated nodopathies. The principal therapeutic classes, their representative agents, and their current approval status are summarized schematically in Figure 4.

*1.*  *Complement inhibitors*
  Complement-mediated mechanisms contribute to demyelination in CIDP. Rilipubart (SAR445088), a humanized monoclonal antibody targeting C1s, is undergoing Phase 2 clinical evaluation in patients with inadequate response to, failure of, or no prior exposure to standard therapies [28]. Interim data indicate disease stabilization or improvement, along with benefits in fatigue, quality of life, and biomarker profiles [28,77]. Additional complement inhibitors under investigation include eculizumab and ravulizumab, which remain in active clinical trials, as well as zilucoplan and GL-2045, which have not yet progressed to clinical testing in CIDP [28].
*2.*  *FcRn antagonists*
  FcRn blockade, which demonstrated efficacy in myasthenia gravis, has recently emerged as a promising therapeutic strategy in CIDP. The pivotal Phase 3 ADHERE trial demonstrated that efgartigimod significantly reduced the risk of relapse compared to placebo, leading to FDA and EMA approval for the treatment of active CIDP following corticosteroid or immunoglobulin therapy. Similar to their application in myasthenia gravis, the use of FcRn antagonists in CIDP is limited by high cost, the requirement for chronic or repeated dosing to sustain IgG suppression, and the non-selective reduction in total IgG. Additionally, long-term safety data remain under investigation. Other FcRn antagonists, such as rozanolixizumab, nipocalimab, and batoclimab, are in late-phase clinical development, although current findings remain inconsistent [77].
*3.*  *B-cell-directed therapies*
  Although B-cells may not be the primary drivers of CIDP, their downstream signaling can amplify inflammatory injury, providing support for the rationale behind B-cell-directed therapies. Among these, anti-CD20 agents have shown promise in CIDP. Rituximab demonstrates efficacy in approximately 60% of patients with refractory disease, particularly in those with IgG4 antibodies to paranodal proteins such as CNTN1, Caspr1, or NF155. Randomized control trials in broader CIDP cohorts are ongoing. Ocrelizumab has also been studied, with case reports indicating prevention of further relapses and, in one case, near-complete resolution of electrophysiological abnormalities. The therapeutic potential of ofatumumab, a fully human anti-CD20 monoclonal antibody, and ublituximab, a chimeric anti-CD20 antibody engineered for enhanced FcγRIII binding, remains to be fully determined. Relapse after anti-CD 20 therapy is hypothesized to be related to the persistence of antibody-producing cells, highlighting the need to target additional B-cell populations. These cells often express CD19 and CD38, making them attractive therapeutic targets. Daratumumab, a human monoclonal antibody targeting CD38, was initially developed for the treatment of multiple myeloma and represents a potential candidate, although there are currently no reports of its use in typical CIDP. Recent reviews indicate that B-cell-directed biologics are costly, are used off-label in CIDP, and are associated with risks including infusion reactions, hypogammaglobulinemia, and serious infections. Therefore, these agents are typically reserved for carefully selected patients who are refractory to standard treatments [28].
  Bruton’s tyrosine kinase (BTK) is essential for B-cell signaling and represents an attractive therapeutic target. BTK inhibitors are being investigated for their potential to reduce inflammation and demyelination in CIDP. Preliminary evidence indicates that these agents may be especially beneficial for patients with refractory disease [77].
*4.*  *Other immunomodulators*
  Proteasome inhibitors (PIs) target long-lived plasma cells that sustain autoantibody production and contribute to treatment resistance or relapse. In a case series of patients with refractory CIDP, bortezomib achieved disease stabilization and both clinical and electrophysiological improvement, sustained for up to one year with minimal systemic toxicity. However, neurotoxicity remains a significant limitation. Second-generation agents, such as carfilzomib and ixazomib, may offer more favorable safety profiles [28].
  T-cell-specific modulators have also been evaluated for their potential to modulate the T-cell component in CIDP. Fingolimod, a sphingosine-1-phosphate receptor modulator with established efficacy in multiple sclerosis, exhibits immunomodulatory effects by depleting naïve and central memory T cells, as well as reducing B memory cells. However, in the large Phase 3 FORCIDP trial involving patients in remission or standard therapy, fingolimod did not demonstrate significant benefit over placebo for the primary endpoint of time to confirmed worsening, nor for secondary or exploratory outcomes [78].
*5.*  *Cell-based and novel therapies*
  In contrast to myasthenia gravis, next-generation CAR-T cell therapies have not yet been applied in CIDP. These approaches are under development to optimize costimulatory domains and improve efficacy while minimizing treatment-related complications. The lack of published reports likely reflects the substantial risks associated with the therapy, as well as the non-life-threatening nature of CIDP [28]. Conversely, autologous hematopoietic stem cell transplantation (AHSCT) has demonstrated potential for durable remission and functional recovery, although controlled trials comparing AHSCT with conventional or emerging therapies are lacking [28].
*6.*  *Special considerations*
  Chronic inflammatory demyelinating polyneuropathy (CIDP) demonstrates a modest male predominance and occurs more frequently in individuals over 50 years of age, with both incidence and prevalence reaching their highest levels in older populations [4]. Age at onset significantly influences both the clinical phenotype and outcomes, as older patients generally experience greater axonal loss and a higher burden of comorbidities, which collectively diminish the likelihood of full recovery and narrow the safety margin for prolonged immunosuppressive therapy. However, most current studies lack sufficient power for sex-stratified analyses, and no consistent sex-specific differences in the efficacy or safety of IVIG, corticosteroids or newer targeted agents have been demonstrated [4,79].
  In antibody-mediated nodopathies such as anti-CNTN1 or anti-Caspr1, intravenous immunoglobulin (IVIG) is frequently ineffective. Rituximab has demonstrated promise in refractory cases; however, some patients fail to respond due to the persistence of long-lived plasma cells, anti-drug antibody formation, or the risk of hypogammaglobulinemia associated with repeated dosing. Emerging evidence suggests that mycophenolate mofetil (MMF) combined with corticosteroids has shown favorable outcomes in small series, though larger studies are needed to establish its role [80]. These therapeutic patterns highlight the importance of antibody identification in guiding treatment decisions. The failure of broad immunosuppressants such as fingolimod in clinical trials for CIDP demonstrates that empirical translation of therapies from other autoimmune diseases is unlikely to succeed unless age-related immune alterations, axonal pathology, and nodal or paranodal mechanisms are adequately addressed [28].

### 3.3. Idiopathic Inflammatory Myopathies

**Figure 5:**
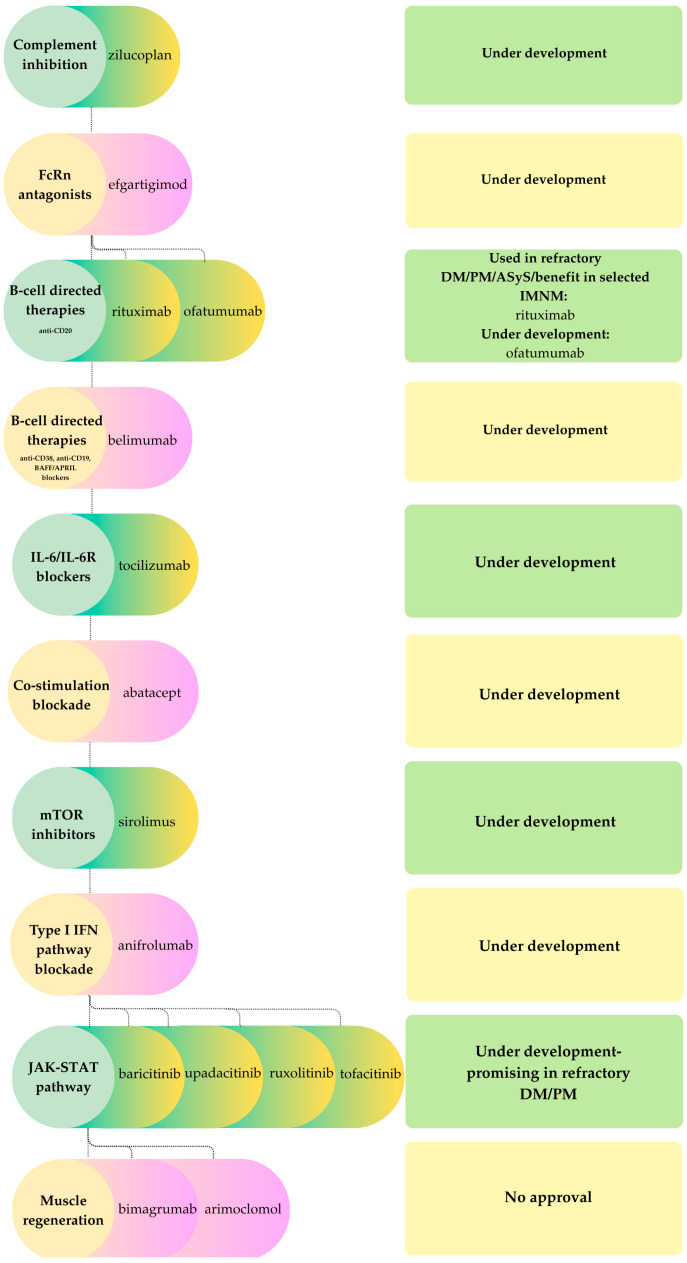
*Drug classes, corresponding representatives and approval status in IIM.* Drug classes are presented with their corresponding representatives, while status boxes summarize which agents are approved for use in idiopathic inflammatory myopathies, which are still under development and which are investigational only; FcRn = neonatal Fc receptor, CD = cluster of differentiation, IL-6/IL-6R = interleukin-6, interleukin-6 receptor, IFN = interferon, mTOR = mechanistic target of rapamycin, BAFF/APRIL = B-cell activating factor/A proliferation-inducing ligand, JAK-STAT = janus kinase-signal transducer and activator of transcription pathway.

Therapeutic responses in idiopathic inflammatory myopathies (IIMs) are closely aligned with the underlying pathogenic mechanisms. Humoral- and complement-mediated subtypes, such as dermatomyositis and immune-mediated necrotizing myopathy, are particularly amenable to B-cell depletion, complement inhibition, and IgG-targeted therapies. In contrast, T-cell dominant polymyositis and the mixed immune-degenerative phenotype of inclusion body myositis (IBM) generally exhibit only modest or heterogenous responses to conventional immunosuppression. Despite these distinctions, systemic immunotherapies, including glucocorticoids, conventional synthetic disease-modifying antirheumatic drugs (DMARDs) such as methotrexate and azathioprine, and intravenous immunoglobulin (IVIG), remain the mainstay of care, although their efficacy is variable and long-term toxicity is a concern [81]. Recent advances in the understanding of humoral, complement, T-cell, and degenerative pathways have facilitated the development of novel agents, which can be categorized by mechanism of action. These include complement inhibition, FcRn antagonism, B-cell depletion, cytokine and interferon blockade, costimulatory modulation, and T-cell-directed therapies, as well as experimental approaches to enhance muscle regeneration and target degenerative processes. In parallel with Figure 3 and Figure 4, Figure 5 summarizes therapeutic categories alongside specific drugs and their approval status.

*1.*  *Complement inhibitors*
  Complement cascade activation contributes to muscle fiber injury in specific IIM subtypes, particularly immune-mediated necrotizing myopathy (IMNM), where pathogenic antibodies (anti-HMGCR, anti-SRP) trigger complement deposition. Despite this, inhibition of the terminal pathway has not been demonstrated to be efficacious in established disease. For example, zilucoplan, a C5 inhibitor, did not improve muscle strength or function in a Phase II placebo-controlled trial. Likewise, in preclinical models, therapeutic C5 blockade after disease onset was ineffective in restoring muscle strength, whereas prophylactic administration prevented disease development. These observations indicate that complement activation in IIM may be a secondary effect rather than a primary pathogenic mechanism [82].
*2.*  *FcRn antagonists*
  Given the proven pathogenic role of IgG autoantibodies in myositis, therapies targeting the neonatal Fc receptor (FcRn) have been explored. Efgartigimod has shown promise in immune-mediated necrotizing myopathy (IMNM): in a recent case series involving refractory IMNM, efgartigimod produced rapid improvements in muscle strength within four weeks. The benefits persisted beyond a single treatment cycle [83], similar to sustained responses observed with FcRn blockade in myasthenia gravis. Although these findings are promising, questions remain regarding the durability of response, as repeated dosing may be necessary to maintain long-term remission. Controlled clinical trials of FcRn antagonists in IIM are required to establish efficacy [83].
*3.*  *B-cell targeted therapies*
  Autoantibody-producing B cells are central to many IIM subtypes, supporting the use of B-cell depletion strategies as a potential treatment option. A systematic review and meta-analysis of 26 studies including patients with dermatomyositis, polymyositis, antisynthetase syndrome, immune-mediated necrotizing myopathy and overlap myositis, but excluding sporadic inclusion body myositis, reported an overall rituximab response rate of about 65%. The efficacy estimate for antisynthetase syndrome was 62%. Severe adverse events and infections occurred in approximately 8% and 2% of patients, respectively. These findings indicate that rituximab is an effective and relatively safe treatment option for refractory, autoantibody-mediated IIM. However, randomized controlled trials are still needed to confirm these results [84]. In IMNM, rituximab and other B-cell directed agents, such as ofatumumab and belimumab, have shown clinical benefit in select treatment-resistant patients [83]. These results bring out the value of depleting B cells or neutralizing B-cell survival factors in autoantibody-mediated IIM. As previously noted in the context of CIDP, B-cell-directed biologics are expensive, predominantly used off-label, and carry risks including hypogammaglobulinemia and serious infections. Comparable considerations are relevant in IIM, where their administration is typically limited to carefully selected, refractory cases pending more comprehensive trial data and long-term safety outcomes.
*4.*  *Other immunomodulators*
  Janus kinase (JAK) inhibitors represent a promising strategy for targeting cytokine signaling in IIM. Agents such as baricitinib, upadacitinib, ruxolitinib, and tofacitinib have demonstrated significant clinical improvements in refractory dermatomyositis (DM) and polymyositis (PM), especially in patients with prominent cutaneous involvement. A recent meta-analysis confirmed both the efficacy and safety of JAK-STAT pathway inhibition in DM and PM, supporting its broader application in myositis management [85].
  IL-6 inhibition. Interleukin-6 (IL-6) is a pro-inflammatory cytokine implicated in the pathogenesis of myositis. Tocilizumab, an anti-IL-6 receptor monoclonal antibody, has shown benefit in both experimental and clinical settings. Two recent case reports described complete responses in anti-synthetase syndrome (ASyS) refractory to conventional therapies, including rituximab, with rapid normalization of muscle strength and systemic improvement. Although additional studies are necessary, these findings indicate that IL-6 inhibition may be a promising therapeutic option for refractory IIM, particularly in ASyS with systemic or articular involvement [86].
  IFN-pathway blockade. Type I interferon (IFN) pathway activation is a characteristic feature in dermatomyositis (DM), prompting evaluation of IFN pathway inhibition as a therapeutic strategy. Anifrolumab, a monoclonal antibody targeting the type I IFN receptor and approved in 2021 for systemic lupus erythematosus, has shown potential in refractory DM. Clinical reports indicate improved control of extramuscular disease, facilitation of glucocorticoid tapering, and reduction in the need for additional immunosuppressive therapy. These findings suggest that systemic IFN blockade may be effective for treatment-resistant DM with multi-organ involvement [87,88].
  Targeting T-cell costimulatory pathways represents another mechanistic approach in IIM. Abatacept, a CTLA-4-Ig fusion protein, inhibits T-cell co-stimulation and addresses dysregulated T-cell activity. It has been evaluated for its ability to reduce T-cell activation in myositis. Although initial reports suggested a benefit in refractory IIM, a 2021 randomized trial did not demonstrate a significant improvement in the overall study population. However, subgroup analyses revealed greater responsiveness in polymyositis (PM) and immune-mediated necrotizing myopathy (IMNM) compared to dermatomyositis, suggesting the potential for selective therapeutic benefit [81].
  Inclusion body myositis (IBM) is characterized by prominent T-cell infiltration and muscle fiber degeneration. The mTOR inhibitor sirolimus (rapamycin) has been investigated for its ability to suppress effector T-cells and promote muscle autophagy. A Phase II trial conducted from 2015 to 2017 demonstrated that sirolimus slowed functional decline in IBM, although the observed benefits were modest. These findings prompted the initiation of a Phase III trial [89].
*5.*  *Cell-based and novel therapies*
  Cellular therapies have been investigated in refractory polymyositis (PM) and dermatomyositis (DM), although clinical experience is limited. Autologous hematopoietic stem cell transplantation (AHSCT) can induce remission by reconstituting the immune system, but carries substantial risks. Mesenchymal stem cell (MSC) transplantation, which does not require myeloablation, has shown encouraging results in DM and PM, and has also led to anecdotal functional improvements in a small number of IBM cases, although clinical studies are scarce [90].
  CD19-directed chimeric antigen receptor T (CAR-T) cell therapy has recently been studied in various IIM subtypes. Preliminary clinical experience indicates that this approach is generally well tolerated and may induce durable remission in select patients. A Phase I/II trial is currently evaluating CABA-201, a fully human anti- CD19 CAR-T cell product, in patients with refractory IIM. In an initial case of IMNM resistant to multiple immunosuppressants, CABA-201 was well tolerated, effectively depleted B cells, and reduced disease-associated autoantibodies without impairing pre-existing humoral immunity, highlighting the potential of this strategy [91]. In conjunction with previous MG studies, these findings indicate that CAR-T therapy can induce profound B-cell depletion and clinical remission in refractory MG and IMNM, with a more favorable safety profile than its use in oncology. However, long-term follow-up and studies involving larger cohorts remain necessary.
*6.*  *Special considerations*
  Within the idiopathic inflammatory myopathy spectrum, sex and age distributions are heterogeneous and influence both clinical phenotype and therapeutic decision-making. Antibody-mediated forms such as dermatomyositis and immune-mediated necrotizing myopathy, typically exhibit a female predominance and most often present in mid- to late adulthood, with statin-associated IMNM occurring more frequently in older individuals [82,83,84]. In contrast, inclusion body myositis is primarily an age-restricted myopathy, usually manifesting after 50 years of age, with a higher frequency in men, and is notable for its resistance to conventional immunosuppressive therapies [89,92,93,94]. Age further impacts comorbidity burden, thereby reducing the safety margin for prolonged high-dose corticosteroid or intensive combination regimens. Although these epidemiological patterns are well established, clinical trials of the aforementioned therapeutic classes generally enroll insufficient numbers of patients to allow for robust sex- or age-stratified analyses, and no consistent differences in efficacy or safety between men and women have been demonstrated to date [82,83,84,85,86,95].
  Given the frequent lack of response to standard immunotherapies in IBM, muscle-targeted strategies have been explored as a potential approach. One such approach involves inhibiting myostatin, a member of the TGF-β family and a negative regulator of skeletal muscle mass. Bimagrumab, a fully human monoclonal antibody that blocks activin type 2 receptors and inhibits myostatin signaling, was found to be safe and increased muscle mass in clinical trials, but did not improve strength, mobility, or walking distance, even with extended treatment duration [92,93]. Another strategy to enhance muscle fiber proteostasis involves arimoclomol, an oral co-inducer of the heat shock response intended to improve protein clearance and reduce cellular stress. However, arimoclomol also failed to demonstrate meaningful clinical benefit. These results suggest that effective therapies for IBM may need to target multiple pathways simultaneously, including degeneration, inflammation, mitochondrial dysfunction, and muscle atrophy [94].

**Table 3:** Principal immune targets and therapeutic landscape across autoimmune neuromuscular disorders.

Drug Class/Mechanistic Target	MG	CIDP	IIM
**Shared across all three pathologies**			
Complement inhibitors	√	√	√
FcRn antagonists	√	√	√
B-cell directed therapies (anti-CD20)	√	√	√
B-cell directed therapies (anti-CD19, anti-CD38, anti-BCMA, BAFF/APRIL blockers)	√	√	√
**Shared across two pathologies**			
IL-6/IL-6R blockers	√	-	√
Co-stimulation blockade	√	-	√
mTOR inhibitors	√	-	√
Conventional immunosuppressants	√	-	√
**MG-specific**			
Thymectomy	√	-	-
Calcineurin inhibitors	√	-	-
CAR-T/CAAR-T therapies	√	-	-
**CIDP-specific**			
Proteasome inhibitors	-	√	-
S1P receptor modulators	-	√	-
**IIM-specific**			
JAK-inhibitors	-	-	√
Type-I-IFN blockade	-	-	√
Muscle regeneration/proteostasis agents	-	-	√
Cell-based therapies (AHSCT, MSC, CAR-T)	-	-	√

MG = myasthenia gravis; CIDP = chronic inflammatory demyelinating polyneuropathy; IIM = idiopathic inflammatory myopathies; FcRn = neonatal Fc receptor; CD = cluster of differentiation (CD19, CD20- B-cell marker; CD38- plasma-cell marker); BCMA = B-cell maturation agent; BAFF = B-cell activating factor; APRIL = A proliferation-inducing ligand; IL-6/IL-6R = interleukin-6/interleukin-6 receptor; mTOR = mechanistic target of rapamycin; CAR-T = chimeric antigen receptor T-cell therapy; CAAR-T = chimeric autoantibody receptor T-cell therapy; S1P = sphingosine-1-phosphate; JAK = janus kinase; IFN = interferon; AHSCT = autologous hematopoietic stem cell transplantation; MSC = mesenchymal stem cell transplantation. √ indicates that therapies in this drug class are available or under clinical investigation for the respective disease.

## 4. Discussion

As highlighted above, although myasthenia gravis, chronic inflammatory demyelinating polyneuropathy, and idiopathic inflammatory myopathies are clinically distinct, they converge on overlapping immune mechanisms that create similar therapeutic approaches. A persistent challenge across these conditions is the lack of reliable biomarkers for patient stratification and therapy selection. Refractory or degenerative forms, such as seronegative myasthenia gravis, resistant chronic inflammatory demyelinating polyneuropathy, and inclusion body myositis, highlight the limitations of current treatments and the need for innovative or cell-based interventions. Advances in diagnostics, biomarker identification, and machine learning are facilitating the development of more effective therapeutic approaches for these disorders.

Electrophysiologic and imaging innovations have advanced the diagnosis and monitoring of these disorders. In myasthenia gravis, single-fiber electromyography (SFEMG), a specialized EMG technique that records action potentials from individual muscle fibers to assess jitter, remains the most sensitive test for detecting defects in neuromuscular transmission. Near-fiber EMG (NFEMG), which uses concentric needle electrodes and signal processing to emphasize potentials from fibers located close to the needle, has recently emerged as a practical alternative, offering similar sensitivity with greater ease of use [96,97]. In chronic inflammatory demyelinating polyneuropathy, traditional nerve conduction studies (NCS) are now supplemented by nerve ultrasound and MRI neurography, which reveal features such as nerve hypertrophy, hyperintensity, and gadolinium enhancement [98,99]. These multimodal imaging protocols are increasingly recommended for both diagnosis and disease monitoring, and may assist in identifying disease variants; however, their prognostic value remains under investigation [98]. In idiopathic inflammatory myopathies, whole-body MRI and quantitative imaging sequences, such as fat fraction mapping and diffusion tensor imaging, enable detection of muscle edema and fatty replacement. Ultrasound techniques, including power Doppler and shear-wave elastography, provide bedside assessment of inflammation [100].

The development of reliable biomarkers remains a critical unmet need across these disorders. In myasthenia gravis, proteomic and transcriptomic analyses have identified molecular subgroups that predict therapeutic response. For instance, patients with high complement activity and acetylcholine receptor positivity benefit from C5 inhibition [101]. MicroRNA and T-cell repertoire studies hold additional promise, as specific microRNA patterns may identify patients likely to respond to corticosteroids, and HLA-T cell combinations may predict responsiveness to B-cell-targeted therapies [102,103]. In chronic inflammatory demyelinating polyneuropathy, serum neurofilament light chain (NfL) has emerged as a promising biomarker, correlating with disease activity and treatment response. As a molecular marker of axonal damage, NfL offers both predictive and prognostic value and may ultimately complement imaging and electrophysiological assessments in routine clinical practice [104]. In idiopathic inflammatory myopathies, biomarker research is clarifying the relationship between molecular pathways and clinical outcomes. Siglec-1 expression reflects disease activity and therapeutic response [105]. Large multi-omics studies have identified subtype-specific signatures, such as interferon and cytokine pathways in dermatomyositis linked to relapse risk, cytoskeletal and extracellular matrix pathways in immune-mediated necrotizing myopathy associated with prognosis, and distinct metabolic alterations in antisynthetase syndrome [106]. These advances indicate that pathway-level biomarkers may soon refine diagnosis and enable personalized therapy.

Recent advances in spatially resolved techniques have enhanced our understanding of autoimmune neuromuscular disorders by linking immune pathways to specific tissue niches in muscle, nerve and the neuromuscular junction. In myasthenia gravis, quantitative immunohistochemistry demonstrates that the terminal complement complex and IgG1 are concentrated at motor endplates, including in some seronegative patients. This identifies a complement-injured niche at the neuromuscular junction and highlights the mechanism of C5 inhibition [107]. In chronic inflammatory demyelinating polyneuropathy and related conditions, multi-omic studies using single-nucleus and spatial transcriptomic profiling have revealed compartment-specific gene expression and immune cell aggregates in the perineurium and endoneurium. These findings indicate that focal nodal and perivascular immune clusters contribute to conduction block and axonal loss [108]. In idiopathic inflammatory myopathies, spatial transcriptomic profiling of juvenile dermatomyositis muscle shows that interferon-stimulated gene expression and mitochondrial stress signatures are concentrated in perifascicular fibers and adjacent capillaries. This provides a molecular explanation for the classic perifascicular interferon-rich lesion pattern observed in histopathology and complement/MHC analyses [109]. Collectively, these spatial datasets suggest that future biomarker strategies and targeted therapies should address not only circulating autoantibodies but also the dominant tissue niche, such as complement-injured endplates in AChR-positive myasthenia gravis, nodal or perineurial immune hubs in chronic inflammatory demyelinating polyneuropathy, or perifascicular interferon-high regions in dermatomyositis.

Machine learning (ML) is being increasingly explored for its potential applications in diagnostic, prognostic, and therapy selection for myasthenia gravis, chronic inflammatory demyelinating polyneuropathy, and idiopathic inflammatory myopathies. In myasthenia gravis, machine learning has been applied to infrared spectroscopy, microbiome profiles, and imaging data, enabling accurate differentiation between patients and healthy controls [110]. Decision-support tools based on machine learning have been developed to predict disease course and guide treatment. For instance, a decision-tree model (C5.0) identified risk factors for intensive care unit admission, allowing clinicians to anticipate clinical deterioration [111]. In chronic inflammatory demyelinating polyneuropathy, although machine learning applications are less developed than in MG, preliminary evidence supports their usefulness. Algorithms applied to electrophysiological datasets have improved diagnostic accuracy and interpretation. One study using SHAP (SHapley Additive exPlanations) analysis validated established criteria and distinguished among three CIDP subtypes, achieving classification accuracy above 80% and providing a valuable decision-support tool [112]. Machine learning has also been used to predict therapeutic response, with unsupervised models integrating clinical and paraclinical variables to stratify patients by pulse steroid response and six-month prognosis [113]. Although still experimental, such models may eventually support personalized treatment selection and outcome prediction once validated in larger, multi-center cohorts. In idiopathic inflammatory myopathies, machine learning algorithms applied to transcriptomic data have improved the discrimination of disease subtypes by leveraging gene expression signatures from muscle biopsies [114]. Machine learning-driven prognostic tools are also emerging, with various algorithms identifying parameters that best correlate with disease outcomes [115]. While still in early development, these approaches demonstrate the potential of artificial intelligence to refine classification, prognosis, and therapy selection in neuromuscular autoimmune disorders.

These priorities define the future trajectory of the field, in which therapeutic innovation, biomarker development, and technological advancements are expected to converge and advance precision medicine for these distinct yet mechanistically linked disorders.

## 5. Conclusions

In the highly diverse spectrum of autoimmune neuromuscular disorders, moving beyond isolated details toward a broader understanding of mechanistic pathogenesis has led to advancements in therapeutic options. Thus, complement inhibitors, FcRn antagonists, and B-cell-depleting therapies have been accurately described in all three pathologies discussed in this review. Beyond that, although still in early stages, the consequences of this deeper understanding have also led to identifying gaps in knowledge that are shared across disorders within this spectrum. There is still much to be explored in the future regarding valid biomarker development or diagnostic techniques, and technological advances, with the involvement of machine learning applications, are expected to contribute significantly. Ultimately, addressing these questions through future validation in multicenter longitudinal cohort studies may indeed constitute the foundation of superior management with only positive consequences for patients.

## Figures and Tables

**Table 1:** Autoantibody targets in myasthenia gravis [8,11,13].

Target of Autoantibodies	Predominant IgG Subclasses	Complement Activation	Key Features
**AChR**	IgG1, IgG3	Yes Classical pathway (C1q → C3b/C4b, C3a/C5a, MAC)	Postsynaptic injury, loss of folds, reduced functional AChRs
**MuSK**	IgG4 (becomes functionally monovalent via Fab-arm exchange)	No Complement-independent mechanism	Inhibits LRP4-MuSK-agrin signaling → disrupts AChR clustering
**LRP4**	IgG1 or IgG2	Can activate complement, but less effectively	Blocks agrin-LRP4-MuSK signaling; lower circulating complement fragments; often milder phenotype
**Agrin**	-	-	Less frequently implicated

AChR = acetylcholine receptor; MuSK = muscle-specific kinase; LRP4 = low-density lipoprotein receptor-related protein 4; MAC = membrane attack complex; Ig = immunoglobulin.

**Table 2:** Clinical and immunological profiles of autoimmune nodopathies in CIDP [23,24].

Nodopathy	Target Antigens	Typical Onset	Key Clinical Features	Antibody Isotypes	Biomarkers/ Monitoring	Notable Associations
**Anti-CNTN1**	Contactin-1 (CNTN1)	Subacute	Predominant motor involvement, ataxia, cranial nerve deficits	IgG4, IgG3	Titers decline with effective therapy; useful for monitoring/relapse prediction	May be associated with nephrotic syndrome
**Anti-Caspr1**	Contactin-associated protein 1 (Caspr1)	Acute/subacute	Tetraparesis, sensory deficits, cranial neuropathies, ataxia, tremor; respiratory failure	IgG4, IgG3	Titers decline with effective therapy; monitoring value	Can be severe; cranial/respiratory involvement
**Anti-NF155**	Neurofascin-155 (paranodal)	Variable	Distal motor weakness, cerebellar-like tremor, ataxia	(often) IgG4	Titers useful for disease monitoring; elevated CSF protein is common	HLA-DRB1*15 association
**Anti-nodal** **neurofascin** **(NF140/186)**	Neurofascin-140 and neurofascin-186	Variable	Often severe; tetraplegia, dysautonomia, cranial nerve involvement, nephrotic syndrome, respiratory compromise	Not specified (IgG subclasses reported variably)	Antibody detection supports diagnosis; severity guides close follow-up	Targets nodal isoforms; severe autonomic/respiratory involvement
**Anti-pan-neurofascin**	Neurofascin-140, neurofascin 155, neurofascin-186	Variable	Severe phenotypes similar to above; multi-system involvement	Not specified (often pathogenic)	Antibody levels for tracking; high vigilance needed	Interferes with node of Ranvier assembly (pathogenicity supported experimentally)

CIDP = chronic inflammatory demyelinating polyneuropathy; CNTN1 = contactin-1; Caspr1 = contactin-associated protein-1; NF = neurofascin; NF155 = neurofascin isoform 155 (paranodal); NF140/186 = neurofascin isoforms 140 and 186 (nodal); CSF = cerebrospinal fluid, HLA = human leukocyte antigen (HLA-DRB*15 allele), IgG = immunoglobulin G (subclasses noted: IgG3, IgG4).

## Data Availability

The raw data supporting the conclusions of this article will be made available by the authors without undue reservation.

## Abbreviation

AChR	acetylcholine receptor
ACR	American College of Rheumatology
ADAPT	Phase 3 trial of efgartigimod in MG
ADHERE	Phase 3 trial of efgartigimod in CIDP
AHSCT/HSCT	autologous hematopoietic stem cell transplantation/hematopoietic stem cell transplantation
AI	artificial intelligence
AIRE	autoimmune regulator
APRIL	A Proliferation-Inducing Ligand
APC	article processing charge
ASyS	antisynthetase syndrome
BAFF	B-cell activating factor
BCMA	B-cell maturation antigen
BLyS	B-lymphocyte stimulator
BTK	Bruton’s tyrosine kinase
CAAR-T	chimeric autoantibody receptor T-cell therapy
CAR-T	chimeric antigen receptor T-cell therapy
Caspr1	contactin-associated protein-1
CD	cluster of differentiation
CIDP	chronic inflammatory demyelinating polyneuropathy
CISP	chronic immune sensory polyradiculopathy
CK	creatine kinase
CNTN1	contactin-1
CSF	cerebrospinal fluid
CTLA-4-Ig	cytotoxic T-lymphocyte-associated protein-4-IgG fusion protein
CXCL13/CCL21	chemokines (thymic hyperplasia context)
DADS	distal acquired demyelinating symmetric neuropathy
DM	dermatomyositis
DMARDs (csDMARDs)	disease-modifying antirheumatic drugs (conventional synthetic)
DTI	diffusion tensor imaging
EAN	experimental autoimmune neuritis
ECG	electrocardiogram
EMA/FDA	European Medicines Agency/U.S. Food and Drug Administration
EMG	electromyography
EULAR	European Alliance of Associations for Rheumatology
FcRIII	Fc gamma receptor III
FcRn	neonatal Fc receptor
FORCIDP	Phase 3 trial of fingolimod in CIDP
GC	glucocorticoids
GL-2045	investigational complement-pathway agent
GRP78/BiP	glucose-regulated protein 78/binding immunoglobulin protein
HLA	human leukocyte antigen
HMGCR	3-hydroxy-3-methylglutaryl-CoA reductase
HSR	heat-shock response
IBM	inclusion body myositis
ICAM/VCAM	intercellular/vascular cell adhesion molecule
IFN/IFNAR	interferon/interferon-α/β receptor
IgG/IgM	immunoglobulin G/M
IIM	idiopathic inflammatory myopathies
IL-6/IL-6R	interleukin-6/interleukin-6 receptor
ILD	interstitial lung disease
IMNM	immune-mediated necrotizing myopathy
ISGs	interferon-stimulated genes
IVIG/SCIG	intravenous/subcutaneous immunoglobulin
JAK-STAT	Janus kinase-signal transducer and activator of transcription
LEMS	Lambert-Eaton myasthenic syndrome
LRP4	low-density lipoprotein receptor-related protein 4
MAC/TCC	membrane attack complex (C5b-9)/terminal complement complex
MADSAM	multifocal acquired demyelinating sensory and motor neuropathy (Lewis-Sumner)
MAG	myelin-associated glycoprotein
MGTX	randomized thymectomy trial in MG
MiRNA	microRNA
ML	machine learning
MMF	mycophenolate mofetil
MPZ/P0	myelin protein zero
MRI	magnetic resonance imaging
MuSK	muscle-specific tyrosine kinase
MxA	myxovirus resistance protein A
NCS	nerve conduction studies
NF/NF140/186/NF155/pan-NF	neurofascin isoforms and pan-neurofascin antibodies
NFEMG/SFEMG	near-fiber EMG/single-fiber EMG
NfL	neurofilament light chain
NMJ	neuromuscular junction
NMOSD	neuromyelitis optica spectrum disorder
NOS2	inducible nitric oxide synthase
OM	overlap myositis
PD-1/PD-L1/PD-L2	programmed death-1 and ligands
PFA/PFN	perifascicular atrophy/perifascicular necrosis
PIs	proteasome inhibitors
PMP22	peripheral myelin protein 22
QoL	quality of life
S1P	sphingosine-1-phosphate (receptor)
SHAP	SHapley Additive exPlanations
SRP	signal recognition particle
TACI	transmembrane activator and CAML interactor
TCR	T-cell receptor
TDP-43	TAR DNA-binding protein 43
Tfh/Tfr	T-follicular helper/T-follicular regulatory cells
TGF-β	transforming growth factor-β
TIM-3	T-cell immunoglobulin and mucin-domain containing-3
TNF	tumor necrosis factor

## References

[1] Bidkar P.U., Satya Prakash M.V.S. (2017). Neuromuscular Disorders. Essentials of Neuroanesthesia.

[2] Shelly S., Mielke M.M., Paul P., Milone M., Tracy J.A., Mills J.R., Klein C.J., Ernste F.C., Mandrekar J., Liewluck T. (2022). Incidence and prevalence of immune-mediated necrotizing myopathy in adults in Olmsted County, Minnesota. Muscle Nerve.

[3] Osman M., Martins K.J.B., Wong K.O., Vu K., Guigue A., Cohen Tervaert J.W., Gniadecki R., Klarenbach S.W. (2023). Incidence and prevalence, and medication use among adults living with dermatomyositis: An Alberta, Canada population-based cohort study. Sci. Rep..

[4] Broers M.C., De Wilde M., Lingsma H.F., Van Der Lei J., Verhamme K.M.C., Jacobs B.C. (2022). Epidemiology of chronic inflammatory demyelinating polyradiculoneuropathy in The Netherlands. J. Peripher. Nerv. Syst..

[5] Miller-Wilson L.-A., Arackal J., Edwards Y., Schwinn J., Rockstein K.E., Venker B., Nowak R.J. (2025). Epidemiology and patient characteristics of the US myasthenia gravis population: Real-world evidence from a large insurance claims database. BMJ Neurol. Open.

[6] Nguyen-Cao T.M., Gelinas D., Griffin R., Mondou E. (2019). Myasthenia gravis: Historical achievements and the “golden age” of clinical trials. J. Neurol. Sci..

[7] Kaminski H.J., Sikorski P., Coronel S.I., Kusner L.L. (2024). Myasthenia gravis: The future is here. J. Clin. Investig..

[8] Yi J.S., Guptill J.T., Stathopoulos P., Nowak R.J., O’Connor K.C. (2018). B cells in the pathophysiology of myasthenia gravis. Muscle Nerve.

[9] Yixian Z., Hai W., Xiuying L., Jichun Y. (2025). Advances in the genetics of myasthenia gravis: Insights from cutting-edge neuroscience research. Front. Med..

[10] Witthayaweerasak J., Rattanalert N., Aui-aree N. (2021). Prognostic factors for conversion to generalization in ocular myasthenia gravis. Medicine.

[11] Le Panse R., Berrih-Aknin S., Kaminski H.J., Kusner L.L. (2018). Immunopathogenesis of Myasthenia Gravis. Myasthenia Gravis and Related Disorders.

[12] Dresser L., Wlodarski R., Rezania K., Soliven B. (2021). Myasthenia Gravis: Epidemiology, Pathophysiology and Clinical Manifestations. J. Clin. Med..

[13] Conti-Fine B.M., Milani M., Kaminski H.J. (2006). Myasthenia gravis: Past, present, and future. J. Clin. Investig..

[14] Michailidou I., Patsiarika A., Kesidou E., Boziki M.K., Parisis D., Bakirtzis C., Chroni E., Grigoriadis N. (2025). The role of complement in the immunopathogenesis of acetylcholine receptor antibody-positive generalized myasthenia gravis: Bystander or key player?. Front. Immunol..

[15] Chuquisana O., Stascheit F., Keller C.W., Pučić-Baković M., Patenaude A.-M., Lauc G., Tzartos S., Wiendl H., Willcox N., Meisel A. (2024). Functional Signature of LRP4 Antibodies in Myasthenia Gravis. Neurol. Neuroimmunol. Neuroinflamm..

[16] Wu Y., Luo J., Garden O.A. (2020). Immunoregulatory Cells in Myasthenia Gravis. Front. Neurol..

[17] Stathopoulos P., Kumar A., Heiden J.A.V., Pascual-Goñi E., Nowak R.J., O’Connor K.C. (2018). Mechanisms underlying B cell immune dysregulation and autoantibody production in MuSK myasthenia gravis. Ann. N. Y. Acad. Sci..

[18] Lazaridis K., Tzartos S.J. (2020). Myasthenia Gravis: Autoantibody Specificities and Their Role in MG Management. Front. Neurol..

[19] Koike H., Nishi R., Ikeda S., Kawagashira Y., Iijima M., Katsuno M., Sobue G. (2018). Ultrastructural mechanisms of macrophage-induced demyelination in CIDP. Neurology.

[20] Quint P., Schroeter C.B., Kohle F., Öztürk M., Meisel A., Tamburrino G., Mausberg A.K., Szepanowski F., Afzali A.M., Fischer K. (2024). Preventing long-term disability in CIDP: The role of timely diagnosis and treatment monitoring in a multicenter CIDP cohort. J. Neurol..

[21] Doneddu P.E., Cocito D., Manganelli F., Fazio R., Briani C., Filosto M., Benedetti L., Mazzeo A., Marfia G.A., Cortese A. (2019). Atypical CIDP: Diagnostic criteria, progression and treatment response. Data from the Italian CIDP Database. J. Neurol. Neurosurg. Psychiatry.

[22] Quinot V., Rostasy K., Höftberger R. (2024). Antibody-Mediated Nodo- and Paranodopathies. J. Clin. Med..

[23] Pascual-Goñi E., Caballero-Ávila M., Querol L. (2024). Antibodies in Autoimmune Neuropathies: What to Test, How to Test, Why to Test. Neurology.

[24] Appeltshauser L., Junghof H., Messinger J., Linke J., Haarmann A., Ayzenberg I., Baka P., Dorst J., Fisse A.L., Grüter T. (2023). Anti-pan-neurofascin antibodies induce subclass-related complement activation and nodo-paranodal damage. Brain.

[25] Vallat J., Mathis S. (2024). Pathology explains various mechanisms of auto-immune inflammatory peripheral neuropathies. Brain Pathol..

[26] Caballero-Ávila M., Martin-Aguilar L., Collet-Vidiella R., Querol L., Pascual-Goñi E. (2025). A pathophysiological and mechanistic review of chronic inflammatory demyelinating polyradiculoneuropathy therapy. Front. Immunol..

[27] Wolbert J., Cheng M.I., Horste G.M.Z., Su M.A. (2020). Deciphering immune mechanisms in chronic inflammatory demyelinating polyneuropathies. JCI Insight.

[28] Mair D., Madi H., Eftimov F., Lunn M.P., Keddie S. (2025). Novel therapies in CIDP. J. Neurol. Neurosurg. Psychiatry.

[29] Gable K.L., Li Y. (2025). Chronic Inflammatory Demyelinating Polyneuropathy: How Pathophysiology Can Guide Treatment. Muscle Nerve.

[30] Schneider-Hohendorf T., Schwab N., Üçeyler N., Göbel K., Sommer C., Wiendl H. (2012). CD8+ T-cell immunity in chronic inflammatory demyelinating polyradiculoneuropathy. Neurology.

[31] Leclair V., Lundberg I.E. (2018). New Myositis Classification Criteria—What We Have Learned Since Bohan and Peter. Curr. Rheumatol. Rep..

[32] Oldroyd A., Chinoy H. (2018). Recent developments in classification criteria and diagnosis guidelines for idiopathic inflammatory myopathies. Curr. Opin. Rheumatol..

[33] Xu S., Hu X., Wang J., Xu Q., Han Z., Zhou H., Gao M. (2023). Polymyositis and dermatomyositis biomarkers. Clin. Chim. Acta.

[34] Silva A.M.S., Campos E.D., Zanoteli E. (2022). Inflammatory myopathies: An update for neurologists. Arq. Neuropsiquiatr..

[35] Ali H., On A., Xing E., Shen C., Werth V.P. (2025). Dermatomyositis: Focus on cutaneous features, etiopathogenetic mechanisms and their implications for treatment. Semin. Immunopathol..

[36] Honda M., Shimizu F., Sato R., Nakamori M. (2024). Contribution of Complement, Microangiopathy and Inflammation in Idiopathic Inflammatory Myopathies. J. Neuromuscul. Dis..

[37] Mammen A.L., Allenbach Y., Stenzel W., Benveniste O., Allenbach Y., Benveniste O., Bleecker J.D., Boyer O., Casciola-Rosen L., Christopher-Stine L. (2020). 239th ENMC International Workshop: Classification of dermatomyositis, Amsterdam, the Netherlands, 14–16 December 2018. Neuromuscul. Disord..

[38] Argyriou A., Horuluoglu B., Galindo-Feria A.S., Diaz-Boada J.S., Sijbranda M., Notarnicola A., Dani L., Van Vollenhoven A., Ramsköld D., Nennesmo I. (2023). Single-cell profiling of muscle-infiltrating T cells in idiopathic inflammatory myopathies. EMBO Mol. Med..

[39] Leclair V., Notarnicola A., Vencovsky J., Lundberg I.E. (2021). Polymyositis: Does it really exist as a distinct clinical subset?. Curr. Opin. Rheumatol..

[40] Cheeti A., Panginikkod S. Dermatomyositis and Polymyositis. https://repository.escholarship.umassmed.edu/server/api/core/bitstreams/2c9a663a-0801-4008-ad38-deec2573cb07/content.

[41] Park Y.-E., Kim D.-S., Kang M., Shin J.-H. (2024). Clinicopathological Reclassification of Idiopathic Inflammatory Myopathy to Match the Serological Results of Myositis-Specific Antibodies. J. Clin. Neurol..

[42] Dalakas M.C. (2011). Review: An update on inflammatory and autoimmune myopathies: Inflammatory myopathies. Neuropathol. Appl. Neurobiol..

[43] Houtman M., Ekholm L., Hesselberg E., Chemin K., Malmström V., Reed A.M., Lundberg I.E., Padyukov L. (2018). T-cell transcriptomics from peripheral blood highlights differences between polymyositis and dermatomyositis patients. Arthritis Res. Ther..

[44] Allenbach Y., Benveniste O., Stenzel W., Boyer O. (2020). Immune-mediated necrotizing myopathy: Clinical features and pathogenesis. Nat. Rev. Rheumatol..

[45] Hoogendijk J.E., Amato A.A., Lecky B.R., Choy E.H., Lundberg I.E., Rose M.R., Vencovsky J., De Visser M., Hughes R.A. (2004). 119th ENMC international workshop: Trial design in adult idiopathic inflammatory myopathies, with the exception of inclusion body myositis, 10–12 October 2003, Naarden, The Netherlands. Neuromuscul. Disord..

[46] Weeding E., Tiniakou E. (2021). Therapeutic Management of Immune-Mediated Necrotizing Myositis. Curr. Treat. Options Rheumatol..

[47] Merlonghi G., Antonini G., Garibaldi M. (2022). Immune-mediated necrotizing myopathy (IMNM): A myopathological challenge. Autoimmun. Rev..

[48] Suzuki S., Nishikawa A., Kuwana M., Nishimura H., Watanabe Y., Nakahara J., Hayashi Y.K., Suzuki N., Nishino I. (2015). Inflammatory myopathy with anti-signal recognition particle antibodies: Case series of 100 patients. Orphanet J. Rare Dis..

[49] Anquetil C., Boyer O., Wesner N., Benveniste O., Allenbach Y. (2019). Myositis-specific autoantibodies, a cornerstone in immune-mediated necrotizing myopathy. Autoimmun. Rev..

[50] Mammen A.L., Chung T., Christopher-Stine L., Rosen P., Rosen A., Doering K.R., Casciola-Rosen L.A. (2011). Autoantibodies against 3-hydroxy-3-methylglutaryl-coenzyme A reductase in patients with statin-associated autoimmune myopathy. Arthritis Rheum..

[51] Allenbach Y., Arouche-Delaperche L., Preusse C., Radbruch H., Butler-Browne G., Champtiaux N., Mariampillai K., Rigolet A., Hufnagl P., Zerbe N. (2018). Necrosis in anti-SRP^+^ and anti-HMGCR^+^ myopathies: Role of autoantibodies and complement. Neurology.

[52] Jacquemin V., Butler-Browne G.S., Furling D., Mouly V. (2007). IL-13 mediates the recruitment of reserve cells for fusion during IGF-1-induced hypertrophy of human myotubes. J. Cell Sci..

[53] Horsley V., Jansen K.M., Mills S.T., Pavlath G.K. (2003). IL-4 Acts as a Myoblast Recruitment Factor during Mammalian Muscle Growth. Cell.

[54] Arouche-Delaperche L., Allenbach Y., Amelin D., Preusse C., Mouly V., Mauhin W., Tchoupou G.D., Drouot L., Boyer O., Stenzel W. (2017). Pathogenic role of anti–signal recognition protein and anti–3-Hydroxy-3-methylglutaryl- C o A reductase antibodies in necrotizing myopathies: Myofiber atrophy and impairment of muscle regeneration in necrotizing autoimmune myopathies. Ann. Neurol..

[55] Tiniakou E., Girgis A., Safaei T.N., Albayda J., Adler B., Paik J.J., Mecoli C.A., Rebman A., Soloski M.J., Christopher-Stine L. (2025). Precise identification and tracking of HMGCR-reactive CD4+ T cells in the target tissue of patients with anti-HMGCR immune-mediated necrotising myopathy. Ann. Rheum. Dis..

[56] Knauss S., Preusse C., Allenbach Y., Leonard-Louis S., Touat M., Fischer N., Radbruch H., Mothes R., Matyash V., Böhmerle W. (2019). PD1 pathway in immune-mediated myopathies: Pathogenesis of dysfunctional T cells revisited. Neurol. Neuroimmunol. Neuroinflamm..

[57] Llansó L., Segarra-Casas A., Domínguez-González C., Malfatti E., Kapetanovic S., Rodríguez-Santiago B., De La Calle O., Blanco R., Dobrescu A., Nascimento-Osorio A. (2024). Absence of Pathogenic Mutations and Strong Association with HLA-DRB1*11:01 in Statin-Naïve Early-Onset Anti-HMGCR Necrotizing Myopathy. Neurol. Neuroimmunol. Neuroinflamm..

[58] O’Hanlon T.P., Rider L.G., Mamyrova G., Targoff I.N., Arnett F.C., Reveille J.D., Carrington M., Gao X., Oddis C.V., Morel P.A. (2006). HLA polymorphisms in African Americans with idiopathic inflammatory myopathy: Allelic profiles distinguish patients with different clinical phenotypes and myositis autoantibodies. Arthritis Rheum..

[59] Ohnuki Y., Suzuki S., Shiina T., Uruha A., Watanabe Y., Suzuki S., Izumi S., Nakahara J., Hamanaka K., Takayama K. (2016). HLA-DRB1 alleles in immune-mediated necrotizing myopathy. Neurology.

[60] Ma X., Bu B.-T. (2022). Anti-SRP immune-mediated necrotizing myopathy: A critical review of current concepts. Front. Immunol..

[61] Greenberg S.A. (2019). Inclusion body myositis: Clinical features and pathogenesis. Nat. Rev. Rheumatol..

[62] Law C., Li H., Bandyopadhyay S. (2021). Coexistence of TDP-43 and C5b-9 staining of muscle in a patient with inclusion body myositis. BMJ Case Rep..

[63] Ngo D.Q., Le S.T., Phan K.H.P., Doan T.T.P., Nguyen L.N.K., Dang M.H., Ly T.T., Phan T.D.A. (2024). Immunohistochemical expression in idiopathic inflammatory myopathies at a single center in Vietnam. J. Pathol. Transl. Med..

[64] Binks S.N.M., Morse I.M., Ashraghi M., Vincent A., Waters P., Leite M.I. (2025). Myasthenia gravis in 2025: Five new things and four hopes for the future. J. Neurol..

[65] Beland B., Storek J., Quartermain L., Hahn C., Pringle C.E., Bourque P.R., Kennah M., Kekre N., Bredeson C., Allan D. (2025). Refractory myasthenia gravis treated with autologous hematopoietic stem cell transplantation. Ann. Clin. Transl. Neurol..

[66] Wolfe G.I., Kaminski H.J., Aban I.B., Minisman G., Kuo H.-C., Marx A., Ströbel P., Mazia C., Oger J., Cea J.G. (2019). Long-term effect of thymectomy plus prednisone versus prednisone alone in patients with non-thymomatous myasthenia gravis: 2-year extension of the MGTX randomised trial. Lancet Neurol..

[67] Gerischer L., Doksani P., Hoffmann S., Meisel A. (2025). New and Emerging Biological Therapies for Myasthenia Gravis: A Focussed Review for Clinical Decision-Making. BioDrugs.

[68] Barrell A. (2024). Efficacy and Safety of Nipocalimab in Patients with Generalised Myasthenia Gravis: Top Line Results from the Double-Blind, Placebo-Controlled, Randomised Phase III Vivacity-MG3 Study. EMJ Neurol..

[69] Jia D., Zhang F., Li H., Shen Y., Jin Z., Shi F.-D., Zhang C. (2024). Responsiveness to Tocilizumab in Anti-Acetylcholine Receptor-Positive Generalized Myasthenia Gravis. Aging Dis..

[70] Cruz J.L., Wolff M.L., Vanderman A.J., Brown J.N. (2015). The emerging role of tacrolimus in myasthenia gravis. Ther. Adv. Neurol. Disord..

[71] Fan Z., Li Z., Shen F., Zhang X., Lei L., Su S., Lu Y., Di L., Wang M., Xu M. (2020). Favorable Effects of Tacrolimus Monotherapy on Myasthenia Gravis Patients. Front. Neurol..

[72] Granit V., Benatar M., Kurtoglu M., Miljković M.D., Chahin N., Sahagian G., Feinberg M.H., Slansky A., Vu T., Jewell C.M. (2023). Safety and clinical activity of autologous RNA chimeric antigen receptor T-cell therapy in myasthenia gravis (MG-001): A prospective, multicentre, open-label, non-randomised phase 1b/2a study. Lancet Neurol..

[73] Haghikia A., Hegelmaier T., Wolleschak D., Böttcher M., Desel C., Borie D., Motte J., Schett G., Schroers R., Gold R. (2023). Anti-CD19 CAR T cells for refractory myasthenia gravis. Lancet Neurol..

[74] Motte J., Sgodzai M., Schneider-Gold C., Steckel N., Mika T., Hegelmaier T., Borie D., Haghikia A., Mougiakakos D., Schroers R. (2024). Treatment of concomitant myasthenia gravis and Lambert-Eaton myasthenic syndrome with autologous CD19-targeted CAR T cells. Neuron.

[75] Kuzmych K., Nachira D., Evoli A., Iorio R., Sassorossi C., Congedo M.T., Spagni G., Senatore A., Calabrese G., Margaritora S. (2025). Surgical and Neurological Outcomes in Robotic Thymectomy for Myasthenic Patients with Thymoma. Life.

[76] Evoli A., Iorio R. (2020). Controversies in Ocular Myasthenia Gravis. Front. Neurol..

[77] Rajabally Y. (2024). Chronic Inflammatory Demyelinating Polyradiculoneuropathy: Current Therapeutic Approaches and Future Outlooks. ImmunoTargets Ther..

[78] Hughes R., Dalakas M.C., Merkies I., Latov N., Léger J.-M., Nobile-Orazio E., Sobue G., Genge A., Cornblath D., Merschhemke M. (2018). Oral fingolimod for chronic inflammatory demyelinating polyradiculoneuropathy (FORCIDP Trial): A double-blind, multicentre, randomised controlled trial. Lancet Neurol..

[79] Hagen K.M., Ousman S.S. (2021). The immune response and aging in chronic inflammatory demyelinating polyradiculoneuropathy. J. Neuroinflamm..

[80] Min Y.G., Ju W., Sung J.-J. (2024). Favorable long-term outcomes of autoimmune nodopathy with mycophenolate mofetil. Front. Neurol..

[81] Aggarwal R., Lundberg I.E., Song Y., Shaibani A., Werth V.P., Maldonado M.A. (2025). Efficacy and Safety of Subcutaneous Abatacept Plus Standard Treatment for Active Idiopathic Inflammatory Myopathy: Phase 3 Randomized Controlled Trial. Arthritis Rheumatol..

[82] Mammen A.L., Amato A.A., Dimachkie M.M., Chinoy H., Hussain Y., Lilleker J.B., Pinal-Fernandez I., Allenbach Y., Boroojerdi B., Vanderkelen M. (2023). Zilucoplan in immune-mediated necrotising myopathy: A phase 2, randomised, double-blind, placebo-controlled, multicentre trial. Lancet Rheumatol..

[83] Yang M., Yuan J., Wang Y., Hao H., Zhang W., Wang Z., Yuan Y., Zhao Y. (2024). Treatment of refractory immune-mediated necrotizing myopathy with efgartigimod. Front. Immunol..

[84] Zhen C., Hou Y., Zhao B., Ma X., Dai T., Yan C. (2022). Efficacy and safety of rituximab treatment in patients with idiopathic inflammatory myopathies: A systematic review and meta-analysis. Front. Immunol..

[85] Ma C., Liu M., Cheng Y., Wang X., Zhao Y., Wang K., Wang W. (2024). Therapeutic efficacy and safety of JAK inhibitors in treating polymyositis/dermatomyositis: A single-arm systemic meta-analysis. Front. Immunol..

[86] Baumann Benvenuti F., Dudler J. (2023). Long-lasting improvement of refractory antisynthetase syndrome with tocilizumab: A report of two cases. RMD Open.

[87] Patel S., Jeurling S., Albayda J., Tiniakou E., Kang J. (2025). Improvement of recalcitrant multisystem disease in dermatomyositis with anifrolumab: A case series. Rheumatology.

[88] Ang P.S., Ezenwa E., Ko K., Hoffman M.D. (2024). Refractory dermatomyositis responsive to anifrolumab. JAAD Case Rep..

[89] Benveniste O., Hogrel J.-Y., Belin L., Annoussamy M., Bachasson D., Rigolet A., Laforet P., Dzangué-Tchoupou G., Salem J.-E., Nguyen L.S. (2021). Sirolimus for treatment of patients with inclusion body myositis: A randomised, double-blind, placebo-controlled, proof-of-concept, phase 2b trial. Lancet Rheumatol..

[90] Gandolfi S., Pileyre B., Drouot L., Dubus I., Auquit-Auckbur I., Martinet J. (2023). Stromal vascular fraction in the treatment of myositis. Cell Death Discov..

[91] Volkov J., Nunez D., Mozaffar T., Stadanlick J., Werner M., Vorndran Z., Ellis A., Williams J., Cicarelli J., Lam Q. (2024). Case study of CD19 CAR T therapy in a subject with immune-mediate necrotizing myopathy treated in the RESET-Myositis phase I/II trial. Mol. Ther..

[92] Amato A.A., Hanna M.G., Machado P.M., Badrising U.A., Chinoy H., Benveniste O., Karanam A.K., Wu M., Tankó L.B., Schubert-Tennigkeit A.A. (2021). Efficacy and Safety of Bimagrumab in Sporadic Inclusion Body Myositis: Long-term Extension of RESILIENT. Neurology.

[93] Hanna M.G., Badrising U.A., Benveniste O., Lloyd T.E., Needham M., Chinoy H., Aoki M., Machado P.M., Liang C., Reardon K.A. (2019). Safety and efficacy of intravenous bimagrumab in inclusion body myositis (RESILIENT): A randomised, double-blind, placebo-controlled phase 2b trial. Lancet Neurol..

[94] Machado P.M., McDermott M.P., Blaettler T., Sundgreen C., Amato A.A., Ciafaloni E., Freimer M., Gibson S.B., Jones S.M., Levine T.D. (2023). Safety and efficacy of arimoclomol for inclusion body myositis: A multicentre, randomised, double-blind, placebo-controlled trial. Lancet Neurol..

[95] Corrales-Selaya C., Prieto-Peña D., Martínez-López D., Benavides-Villanueva F., Blanco R. (2025). Epidemiology of Dermatomyositis and Other Idiopathic Inflammatory Myopathies in Northern Spain. Biomedicines.

[96] Sanders D.B., Kouyoumdjian J.A., Stålberg E.V. (2022). Single fiber electromyography and measuring jitter with concentric needle electrodes. Muscle Nerve.

[97] Mandeville R., Patterson A., Luk J., Eleanore A., Garnés-Camarena O., Stashuk D. (2025). Near Fiber Electromyography in the Diagnosis of Myasthenia Gravis: NFEMG in MG. RRNMF Neuromuscul. J..

[98] Tozza S., Cassano E., Erra C., Muto M., Habetswallner F., Manganelli F. (2025). Role of Imaging in Chronic Inflammatory Demyelinating Polyneuropathy: A Systematic Review. Eur. J. Neurol..

[99] Su X., Kong X., Alwalid O., Wang J., Zhang H., Lu Z., Zheng C. (2021). Multisequence Quantitative Magnetic Resonance Neurography of Brachial and Lumbosacral Plexus in Chronic Inflammatory Demyelinating Polyneuropathy. Front. Neurosci..

[100] Zubair A.S., Salam S., Dimachkie M.M., Machado P.M., Roy B. (2023). Imaging biomarkers in the idiopathic inflammatory myopathies. Front. Neurol..

[101] Nelke C., Schroeter C.B., Barman S., Stascheit F., Masanneck L., Theissen L., Huntemann N., Walli S., Cengiz D., Dobelmann V. (2024). Identification of disease phenotypes in acetylcholine receptor-antibody myasthenia gravis using proteomics-based consensus clustering. eBioMedicine.

[102] Balistreri C.R., Vinciguerra C., Magro D., Di Stefano V., Monastero R. (2024). Towards personalized management of myasthenia gravis phenotypes: From the role of multi-omics to the emerging biomarkers and therapeutic targets. Autoimmun. Rev..

[103] Chung C.-C., Wu I.-C., Bamodu O.A., Hong C.-T., Chiu H.-C. Machine Learning in Myasthenia Gravis: A Systematic Review of Prognostic Models and AI-Assisted Clinical Assessments. https://www.mdpi.com/2075-4418/15/16/2044.

[104] Oeztuerk M., Henes A., Schroeter C.B., Nelke C., Quint P., Theissen L., Meuth S.G., Ruck T. (2023). Current Biomarker Strategies in Autoimmune Neuromuscular Diseases. Cells.

[105] Kamperman R.G., Veldkamp S.R., Evers S.W., Lim J., Van Schaik I., Van Royen-Kerkhof A., Van Wijk F., Van Der Kooi A.J., Jansen M., Raaphorst J. (2025). Type I interferon biomarker in idiopathic inflammatory myopathies: Associations of Siglec-1 with disease activity and treatment response. Rheumatology.

[106] Xiao Y., Xie S., Liu Y., Jiang Y., Li H., Zhang H., Zuo X., Luo H., Zhu H. (2025). Multiomics analysis uncovers subtype-specific mechanisms and biomarkers in idiopathic inflammatory myopathies. Ann. Rheum. Dis..

[107] Meyer Zu Hörste G., Gross C.C., Klotz L., Schwab N., Wiendl H. (2020). Next-Generation Neuroimmunology: New Technologies to Understand Central Nervous System Autoimmunity. Trends Immunol..

[108] Heming M., Börsch A.-L., Wolbert J., Thomas C., Mausberg A.K., Szepanowski F., Eggert B., Lu I.-N., Tietz J., Dienhart F. (2025). Multi-omic identification of perineurial hyperplasia and lipid-associated nerve macrophages in human polyneuropathies. Nat. Commun..

[109] Syntakas A.E., Kartawinata M., Evans N.M.L., Nguyen H.D., Papadopoulou C., Obaidi M.A., Pilkington C., Glackin Y., Mahony C.B., Croft A.P. (2025). Spatial transcriptomic analysis of muscle biopsy from patients with treatment-naive juvenile dermatomyositis reveals mitochondrial abnormalities despite disease-related interferon-driven signature. Ann. Rheum. Dis..

[110] Chang C.-C., Liu T.-C., Lu C.-J., Chiu H.-C., Lin W.-N. (2023). Machine learning strategy for identifying altered gut microbiomes for diagnostic screening in myasthenia gravis. Front. Microbiol..

[111] Chang C.-C., Yeh J.-H., Chiu H.-C., Chen Y.-M., Jhou M.-J., Liu T.-C., Lu C.-J. (2022). Utilization of Decision Tree Algorithms for Supporting the Prediction of Intensive Care Unit Admission of Myasthenia Gravis: A Machine Learning-Based Approach. J. Pers. Med..

[112] Ballanti S., Liuzzi P., Mattiolo P.L., Scarpino M., Matà S., Hakiki B., Cecchi F., Oddo C.M., Mannini A., Grippo A. (2025). Electrophysiological-based automatic subgroups diagnosis of patients with chronic dysimmune polyneuropathies. J. NeuroEng. Rehabil..

[113] Chang C., Ro L., Lyu R., Kuo H., Liao M., Wu Y., Chen C., Chang H., Weng Y., Huang C. (2022). Establishment of a new classification system for chronic inflammatory demyelinating polyneuropathy based on unsupervised machine learning. Muscle Nerve.

[114] McLeish E., Slater N., Mastaglia F.L., Needham M., Coudert J.D. (2023). From data to diagnosis: How machine learning is revolutionizing biomarker discovery in idiopathic inflammatory myopathies. Brief. Bioinform..

[115] Danieli M.G., Paladini A., Longhi E., Tonacci A., Gangemi S. (2023). A machine learning analysis to evaluate the outcome measures in inflammatory myopathies. Autoimmun. Rev..

